# Fibroblast-derived extracellular vesicles as trackable efficient transporters of an experimental nanodrug with fibrotic heart and lung targeting

**DOI:** 10.7150/thno.85409

**Published:** 2024-01-01

**Authors:** Jorge RuizdelRio, Gabriela Guedes, Danielle Novillo, Elena Lecue, Ana Palanca, Aitziber L. Cortajarena, Ana V. Villar

**Affiliations:** 1 Instituto de Biomedicina y Biotecnología de Cantabria (IBBTEC), Consejo Superior de Investigaciones Científicas (CSIC)-Universidad de Cantabria (UC), Santander, Spain.; 2 Center for Cooperative Research in Biomaterials (CIC biomaGUNE), Basque Research and Technology Alliance (BRTA), Paseo de Miramón 194, Donostia-San Sebastián 20014, Spain.; 3 University of the Basque Country (UPV/EHU), 48940 Leioa, Spain.; 4 Departamento de Anatomía y Biología Celular, Universidad de Cantabria, Santander, Spain.; 5 Ikerbasque, Basque Foundation for Science, 48009 Bilbao, Spain.; 6 Departamento de Fisiología y Farmacología, Facultad de Medicina, Universidad de Cantabria, Santander, Spain.

**Keywords:** biological antifibrotic nanocarriers, fibrosis, protein-nanomaterial hybrids, extracellular vesicles, *in vivo* imaging

## Abstract

The discovery of extracellular vesicles (EVs) as efficient exogenous biotransporters of therapeutic agents into cells across biological membranes is an exciting emerging field. Especially the potential of EVs as targeted delivery systems for diseases with selective treatments, such as fibrosis, whose treatment causes side effects in other organs not involved in the disease. Methods: In this study, we collected embryonic fibroblast-derived EVs from two different centrifugation fractions, 10 K g and 100 K g fractions from a NIH-3T3 cell line loaded with an experimental drug. Mice with fibrotic hearts and lungs were obtained by administration of angiotensin II. We generated fluorescent EVs and bioluminescent drug to observe their accumulation by colocalization of their signals in fibrotic heart and lung. The biodistribution of the drug in various organs was obtained by detecting the Au present in the drug nanostructure. Results: The drug-loaded EVs successfully reduced fibrosis in pathological fibroblasts *in vitro*, and modified the biodistribution of the experimental drug, enabling it to reach the target organs *in vivo*. We described the pre-analytical characteristics of EVs related to physical variables, culture and harvesting conditions, crucial for their *in vivo* application as nanotransporters using a previously validated protein-based antifibrotic drug. The results showed the colocalization of EVs and the experimental drug *in vivo* and *ex vivo* and the efficient reduction of fibrosis *in vitro*. This work demonstrates that 10K-EVs and 100K-EVs derived from fibroblasts can act as effective biotransporters for targeted drug delivery to profibrotic fibroblasts, lungs, or heart.

**Conclusion:** We observed that fibroblast-derived 10K-EVs and 100K-EVs are useful biotransporters encapsulating a new generation drug leading to a reduction of fibrosis in profibrotic fibroblasts *in vitro*. In addition, drug containing EVs were shown to reach fibrotic heart and lungs *in vivo*, enhancing free drug biodistribution.

## Introduction

According to the International Society for Extracellular Vesicles (ISEV), extracellular vesicles (EVs) are defined as nanometric particles containing a lipid bilayer that are naturally released by cells to transport their contents. Cell-to-cell communication via EVs can occur both autologously and heterologously linking cells involved in the pathophysiology of different organs. EVs can be divided into three main types: 1) exosomes are originally formed as intraluminal vesicles (ILVs) from multivesicular bodies (MVBs) and with a diameter of approximately 50-150 nm 1; 2) ectosomes or microparticles are generated by direct budding outside the plasma membrane and can have a diameter of up to 500 nm; and 3) apoptotic bodies, that together with ectosomes and oncosomes, can be larger with a diameter of up to 5 μm 2-4. The different sizes and intracellular origins of EVs could affect their behaviors as both natural and exogenous biotransporters and therefore their potential clinical application.

These non-replicative vesicles are difficult to purify due to their heterogeneity in size and markers, unless markers of subcellular origin can be established 5. However, fractions of EVs can be identified based on their size range or complexity when purified using serial centrifugations. Here, we isolated two distinct fractions of EVs corresponding to centrifugations at 10,000 g and at 100,000 g. Labeling of EVs enables their visualization and characterization *in vitro* and *in vivo* 6. There are several successful methods of fluorescence labeling of EVs including insertion of myristoylation, palmitoylation, prenylation sequences, to the fluorescent protein tags 7-8. Prenylation motifs, present in most Ras family members allows targeting of protein to cell membrane 9. We have genetically engineered fibroblasts expressing a prenylated far-red fluorescent protein from which fluorescent EVs suitable for fluorescence analysis were isolated 10.

EVs have been defined as a therapeutic strategy or biomarker in various diseases including cardiac 11 and pulmonary 12 pathologies. The ability of EVs to be generated, secreted, and internalized into cells in response to stress or damage signals, directed by growth factors and cytokines, makes them a perfect communicator between lung and heart 13. Therefore, they have the potential to act as biotransporters of drugs to these highly irrigated organs. For example, in the heart, EVs are already considered an innate drug delivery system 14 since their fibroblasts, a non-myocytic cell type, maintain homeostasis through constant communication with themselves, cardiomyocytes or endothelial cells, especially in stress situations such as hypoxia, infection, inflammation, or fibrosis 15.

We have utilized a model of systemic fibrosis via administration of angiotensin II (Ang II) a potent vasopressor peptide in the pathogenesis of cardiac remodeling 16 and pulmonary hypertension 17. In the case of the heart, if it is affected by a chronic cardiovascular injury (valve narrowing, cardiomyopathies, tissue necrosis, arterial hypertension, etc.) it will develop fibrosis and hypertrophy as remodeling mechanisms to restore or maintain function 18. These remodeling features are beneficial in the short term, but in the long term, uncontrolled myocardial fibrosis produced mainly by fibroblasts (target cells), becomes highly damaging and can cause organ failure 19. Organ failure could be a consequence of cardiac fibrosis due to increased stiffness and decreased distensibility of cardiac tissue. Patients with less fibrosis experienced marked improvement of diseases, such as aortic stenosis after valve replacement, compared to those with high levels of fibrosis, with correlations between left ventricular dysfunction, ventricular fibrosis and worse prognosis 20. Due to the high level of side effects, there is currently no approved antifibrotic treatment for fibrotic patients of cardiovascular etiology 21. Clinical treatments to reduce fibrosis of cardiovascular patients are not antifibrotic drugs but antihypertensive agents such as angiotensin-converting enzyme (ACE) inhibitors and angiotensin II receptor antagonists (ARA-II) 22 or drugs against hypercholesterolemia such as statins 23, but in no case are they designed to reduce fibrosis itself.

In the case of the lung, acute lung injury (ALI), acute respiratory distress syndrome (ARDS), chronic obstructive pulmonary disease (COPD) and idiopathic pulmonary fibrosis (IPF) are examples of lung diseases involving reparative processes. When pathophysiological alteration of the lung occurs, cellular components of the microvascular endothelium, alveolar epithelium, and pulmonary interstitium initiate their organ remodeling process. Proliferating fibroblasts are part of the remodeling process and can produce large amounts of profibrotic proteins that accumulate in the interstitial space 24.

We have recently demonstrated that a promising protein-nanomaterial hybrid (CTPRAu), a fluorescent gold nanocluster stabilized by an engineered Consensus Tetratricopeptide Repeat (CTPR) protein and containing an Hsp90-inhibiting module (CTPR390), showed efficient antifibrotic capabilities 25. Moreover, using high-resolution atomic imaging it was possible to locate this antifibrotic drug in non-apoptotic vesicles inside fibroblasts 26. This observation indicates that fibroblasts naturally transport CTPRAu as part of endocytic trafficking events, including nutrient uptake, signaling from cell surface receptors, or acquisition of new proteins.

Considering the natural disposition of CTPRAu towards cell uptake and with the aim of exploring the possibility of using a biological carrier to target active fibroblasts in profibrotic cellular environments, we encapsulated CTPRAu into healthy fibroblasts derived EVs to study its antifibrotic potential on profibrotic fibroblasts. Previous studies have shown that CTPRAu primarily accumulates in organs associated with drug elimination, however, it achieves a long-term accumulation (up to 1 week) in fibrotic organs 25. In this way, by encapsulating CTPRAu in EVs, we intended to alter its biodistribution pattern, enabling it to reach fibrotic sites *in vivo* in a short amount of time.

## Results

### Physicochemical differences of 10K and 100K fractions containing fluorescent EVs derived from genetically modified NIH-3T3 fibroblasts

**Figure 1:**
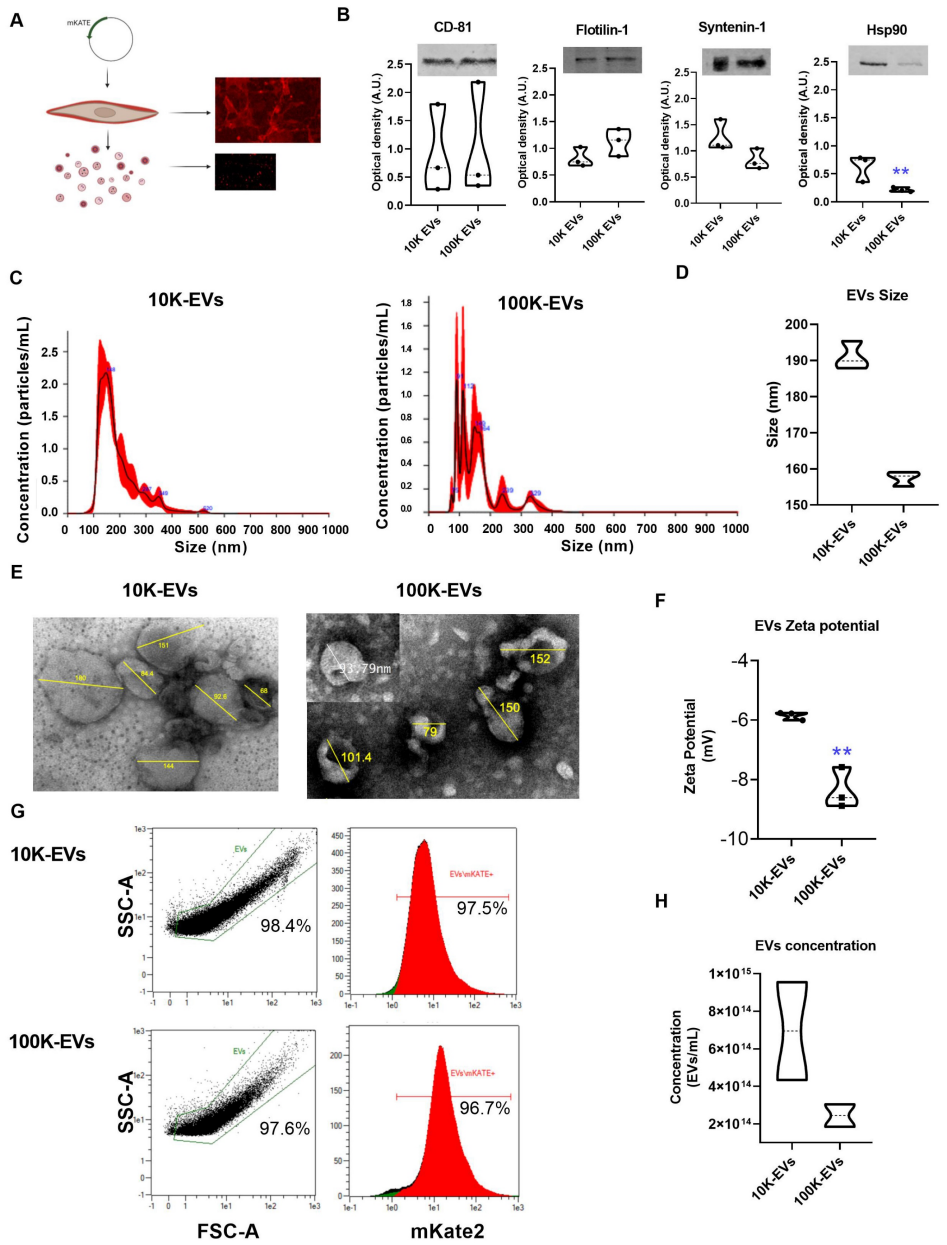
**The 10K and 100K fractions extracted from genetically engineered NIH-3T3 embryonic fibroblasts (mKate2 NIH-3T3) contain EVs. A**) Drawing depicting the insertion of the m-Kate plasmid into fibroblasts and confocal images of the final fluorescent products, fluorescent NIH-3T3 mKate2 fibroblasts and their derived fluorescent mKate2 EVs. **B**) Representative images of EV WBs and graphs showing the signal intensity analysis of the WBs (n = 3) of the membrane markers CD-81 and Flotilin-1 and the soluble markers Syntenin-1 and Hsp90 (**p < 0.005) belonging to m-Kate-NIH-3T3 10K and 100K EVs. **C**) NTA measurements of the concentration and size distribution of usual polydisperse populations of ultracentrifuged EVs. MKate2-NIH-3T3 10K-EVs (left) and mKate2-NIH-3T3 100K-EVs (right); in red the standard deviation of the mean value of the measurements (n = 4). **D**) NTA mean size of the particles under study n = 4 per type of EV (10K and 100K).** E**) Representative transmission electron microscopy images of extracellular vesicles (10K-EVs and 100K-EVs) obtained from mKate2 NIH-3T3 cells by centrifugation. **F**) Charge measurements at a distance from the particle (zeta potential) in mV for 10K-EVs and 100K-EVs (n = 3), **p < 0.005. **G**) Representative images of the detection of mKate2 10K-EVs and mKate2 100K-EVs by flow cytometry. The graphs are the singlet gate analysis of a dot plot of forward scatter versus side scatter in the 645 nm fluorescent channel (mKate2). The graphs indicate the percentages of EVs detected from the total number of particles in the samples (left panels) and the percentages of mKate2 EVs from the total number of EVs in the samples (right panels). **H**) NTA Total concentration of EVs/mL (10K-EVs and 100K-EVs) extracted from 12x10^8^ mKate2 NIH-3T3 cells.

The NIH-3T3 cell line of embryonic fibroblasts is a source of EVs able to interact with other cells and communicate signals important for their homeostasis 27. Our first objective was to develop a cell line designed to express and anchor the mKate2 fluorescent tag on the membranes of EVs. EVs derived from genetically modified NIH-3T3 fibroblasts allow consistent and easy visualization of EVs at a maximum emission of 635 nm. To this end, we fused the red fluorescent protein mKate2 to the C-terminal end of the CAAX motif where C is Cys; AA are the aliphatic amino acids Val and Leu; and X is Ser (Figure S1A). The CAAX box is recognized by endogenous farnesyltransferase which subsequently transfers a farnesyl group to the thiol group of Cys. Farnesylation of mKate2-Cys-Val-Leu-Ser protein increases its hydrophobicity resulting in its anchoring to the cell membrane 8. A schematic representation of the mKate2-CAAX vector used for cell line generation is displayed in Figure S1A-B. We obtained images (Figure S1C) and proliferation curves (Figure S1D) of cells with puromycin selection at 0, 1, 2.5, and 5 ng/mL 3 days after transfection with mKate2 vector, and it was observed that 1 ng/mL of puromycin is sufficient to eliminate NIH-3T3 from the culture. After mKate2 NIH-3T3 cell line expressing membrane bound mKate2 was established, the cell viability was measured (Figure S1E). In addition, the cell surface expression of mKate2 was verified through fluorescence studies (Figure S1F). Thus, we established a new genetically engineered NIH-3T3 fibroblasts stable cell line (mKate2 NIH-3T3) that produces fluorescently labeled EVs as shown in Figure 1A. To obtain EVs, mKate2 NIH-3T3 cells were cultured with media supplemented with depleted FBS. Cell supernatant was ultracentrifuged at 10 Kg and 100 Kg (after removing cell debris in a centrifugation at 2,000 g). The use of EVs-free PBS served to define the reliable area of detection of mKate2 EVs in samples with 1:10 dilution, we discarded the PBS-derived powder and selected the EVs zone by cytometry (Figure S2A). We observed that serial dilutions of the EVs samples presented a decrease in counts in the detection by cytometry with a correct signal/sample ratio at 1:10 and 1:100 dilutions (Figure S2B), thus these working dilutions were selected for mKate2 EVs detection. To demonstrate that mKate2 NIH-3T3 supernatant fractions contained EVs, we first checked for the presence of membrane and soluble EV markers by western blot. The EV membrane markers CD-81 and Flotilin-1 and the EV soluble markers, Syntenin-1 and Heat Shock Protein 90 (Hsp90), a protein related to TGFβ signaling cascade in fibroblasts 16, 28, were detected in both 10K and 100K fractions (Figure 1B). Henceforth, we will refer to fractions as 10K-EVs and 100K-EVs respectively. Interestingly, Hsp90 was significantly more abundant in the 10K fraction than in the 100K fraction (signal intensity was normalized to the loading marker), whereas there was no significant difference in the levels of Syntenin-1, CD-81, and Flotilin-1 between the two fractions (Figure 1B). Nanoparticle Tracking Analysis (NTA) was used to measure the EVs diameter in the 10K-EV and 100K-EV fractions, obtaining the size of the particles and particle number from the scattered light in a distillate water suspension. The most abundant populations of 10K-EVs and 100K-EVs showed a diameter of 148.0 ± 15.0 nm and 91.0 ± 11.0 nm respectively (Figure 1C); with mean values of 189.9 ± 74.3 nm and 158.6 ± 90.4 nm respectively (Figure 1D). We further characterized the 10K-EVs and 100K-EVs fractions by transmission electron microscopy (TEM) by taking representative images of EVs with a diameter range similar to that of the most abundant populations and to populations that represent the average diameters in each of the fractions (Figure 1E). TEM images showed that both fractions of EVs present a spherical morphology. We investigated any charge differences between the 10K-EV and the 100K-EV fractions by measuring the zeta potential of each fraction. mKate2 10K-EVs exhibited a negative zeta potential of -5.8 ± 0.1 mV, whereas mKate2100K-EVs exhibited a significantly (**p < 0.005) more negative zeta potential of -8.4 ± 0.7 mV (Figure 1F). These results indicated the presence of two different fractions (10K-EVs and 100K-EVs) in terms of particle distribution, size and charge.

Finally, we used an adapted Macs protocol (see methods section) to determine sample labeling efficiency by flow cytometry (Figure S2A). Data obtained below the forward scatter (FSC-A) value of 1e1 were sidelined from any given side scatter value (SSC-A). It was found that 97.5% of the 10K-EVs fraction and 96.7% of the 100K-EVs fraction was fluorescently labeled with mKate2. (Figure 1G). We checked the stability of mKate2 10K-EVs and mKate2 100K-EVs under various temperature and serum experimental conditions. We incubated both types of EVs in depleted FBS at 37 ºC for 1 h (Figure S2C), 2 h (Figure S2D) and 7 days (Figure S2E). By flow cytometry, we observed that the complexity of both EVs samples was maintained, in contrast to broken-sonicated EVs as a negative control (Figure S2F). Signal from EVs-depleted-FBS serum (Figure S2G) and from EVs-depleted-PBS (Figure S2H) were also used as negative controls.

These above results indicate the successful labeling of EVs through insertion of mKate2 into their membranes and their stability. The number of mKate2 EVs obtained per mL of fibroblast supernatant under the conditions of the study (mKate2 NIH-3T3 cultured for five days in depleted medium) was measured by NTA. The number of EVs/mL obtained from 12x10^8^ cells was 7.0x10^14^ ± 4.0 x10^10^ EVs/mL for 10K-EVs and 2.5x10^14^ ± 8.5x10^13^ EVs/mL for 100K-EVs as shown in Figure 1H. MKate2 EVs exhibited the same size and Z-potential of regular NIH-3T3 EVs (Figure 1F). These results demonstrated the presence of mKate2 EVs in both 10K and 100K fractions, as well as the differences in size, charge, and concentration between mKate2 10K-EVs and mKate2 100K-EVs, but not in comparison with the 10K and 100K fractions of EVs from normal NIH-3T3 cells.

### Pathological fibroblasts incorporate EVs derived from healthy mKate2 NIH-3T3 cells

**Figure 2:**
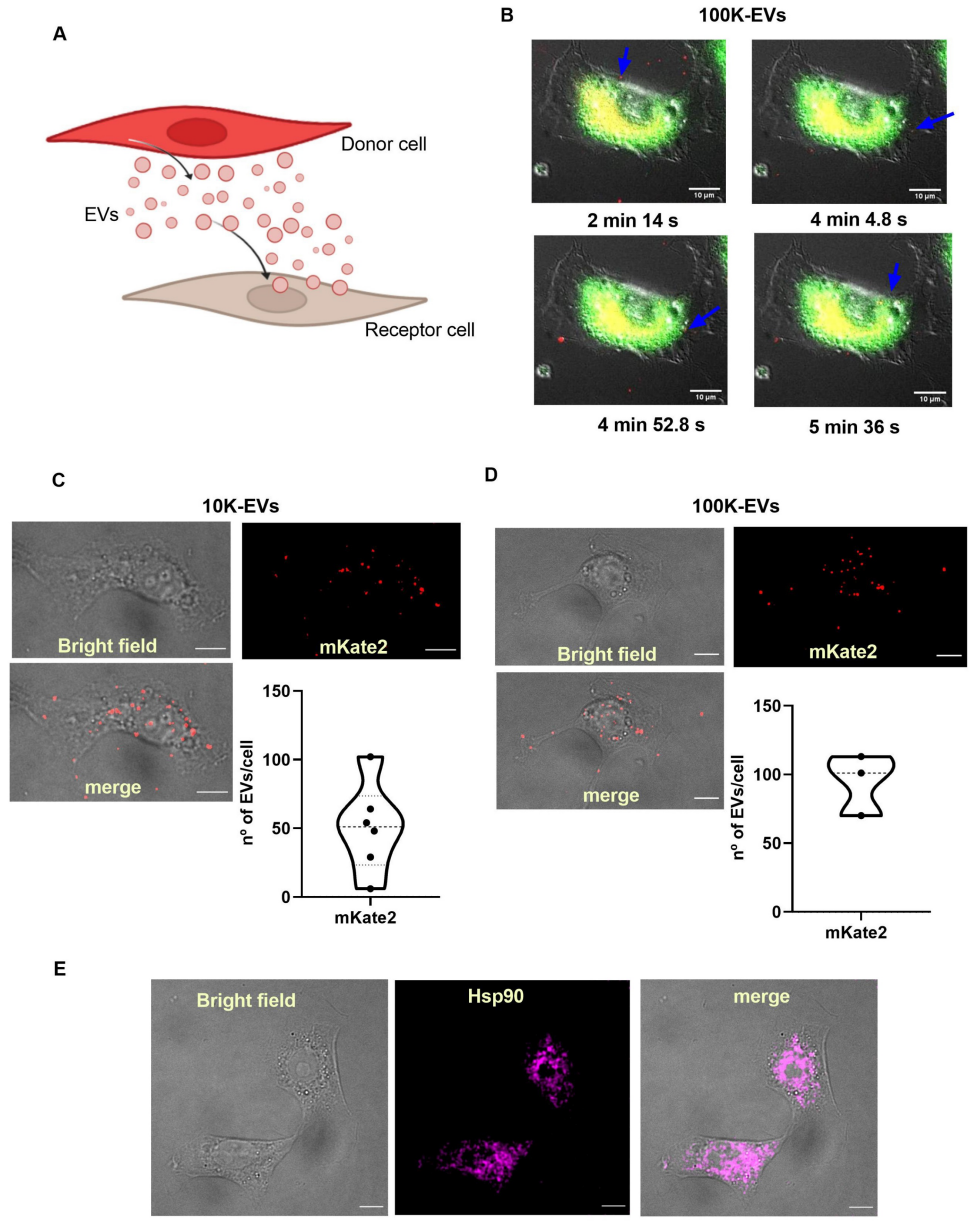
** Internalization of mKate2 10K-EVs and mKate2 100K-EVs in NIH-3T3 profibrotic embryonic fibroblasts. A**) Vignette depicting the secretion of mKate2 EVs from NIH-3T3 mKate2 cells (Donor cells) and their posterior internalization into profibrotic NIH-3T3 cells (Receptor cells). **B**) Live-cell images showing mKate2 100K-EVs (red spots) internalized into profibrotic NIH-3T3 cells, stained in green. Images were captured every 5 s for a total time of 10 min live-cell imaging. Blue arrows point to possible single internalization events. Scale bar: 10 μm. **C,D)** 4 panels images related to TGFβ (1 ng/mL) treated NIH-3T3 cell for 36 h, visualized 10 min after EVs administration of (**C**) 7.0x10^11^ ± 4.0x10^11^ mKate2 10K-EVs/mL or (**D**) 2.5x10^11^ ± 8.5x10^10^ mKate2 100K-EVs/mL: - brightfield; - confocal microscopy image showing intrinsic fluorescence of mKate2 EVs (red dots); overlay of brightfield and fluorescence of mKate2 EVs; graphs showing the number of mKate2 EVs fluorescent dot count/cell using ImageJ software. n = 3-6 independent cells from cell culture (10x10^6^ cells). **E**) Representative images of NIH-3T3 cell treated with TGFβ (1 ng/mL), from left to right: - bright field; - immunohistochemistry image visualized with confocal microscopy of Hsp90αβ detection; merge of same bright field and immunohistochemistry images.

Based on the ability of fibroblasts to secrete and capture EVs 29, our hypothesis advocates that EVs can be used as effective biological nanocarriers to reach target fibrotic fibroblasts *in vitro*. A well-established method for the activation of the synthesis of profibrotic molecules in fibroblasts is the administration of TGFβ, the main cytokine responsible for triggering profibrotic signaling pathways 30. To demonstrate that EVs from basal (healthy) fibroblasts are useful in reaching and internalizing TGFβ-activated (fibrotic) fibroblasts, we separately administered mKate2 10K-EVs and mKate2-100K EVs to such pathological profibrotic cells (Figure 2A). To activate their profibrotic stage, NIH-3T3 cells were treated with TGFβ (1.0 ng/mL) as previously described 16. We proceeded to perform a series of *in vivo* cellular assays to visualize the cellular uptake of mKate2 EVs in TGFβ-activated fibroblasts. We visualized the fluorescent signal of mKate2 EVs purified from basal mKate2 NIH-3T3 fibroblasts after their addition to TGFβ-activated NIH-3T3 fibroblasts. The epifluorescence signal was detected both on the fibroblast membrane (as an indicator that fluorescent EV were entering/passing the cell membrane) and in the conditioned medium of these cells 2-6 min after EV addition. Figure 2B showed a representative image of internalization of mKate2 100K-EVs into NIH-3T3 cells captured during the first min after EVs addition. Fusion of bright field and fluorescence images from confocal microscopy at longer times after EVs addition (10 min) showed localization of fluorescent mKate2 10K-EVs and mKate2 100K-EVs within fibroblasts (Figure 2C-D). The total number of particles incorporated per cell were quantify by identification of the fluorescent dye on the mKate2 protein in randomly selected cells (n = 3-6) (Figure 2C-D). The number of fluorescent particles located inside the cells were quantified by applying a median filter and setting a threshold at the maximum value minus the noise tolerance (see methods section). The fluorescent dots shown, correspond to EVs, which are quantified using ImageJ software (Figure 2C-D). The number of internalized EVs per cell was 50.5 ± 32.5 mKate2-10K EVs/cell and 94.7 ± 22.2 mKate2-100K EVs/cell, 10 min after EVs administration of 7.0x10^11^ ± 4.0 x10^11^ EVs/mL for mKate2 10K-EVs and 2.5x10^11^ ± 8.5x10^10^/mL mKate2 100K-EVs, to 10x10^6^ profibrotic fibrotic NIH-3T3 fibroblasts (Figure 2C-D).

Heat shock protein 90 (Hsp90) is a natural partner of the TGFβ signaling cascade, whose chemical inhibition reduces collagen production (fibrosis) 28. Recently, we demonstrated that inhibition by nanotherapies such as an engineered protein inhibitor (CTPR390) reduces fibrosis *in vitro* and *in vivo* 16. Figure 1B western blots showed the presence of Hsp90 in fibroblast-derived EVs, as a normal trafficking mechanism of a ubiquitous chaperone 16, 28, 31. Hsp90 presence inside NIH-3T3 TGFβ-activated fibroblasts was evaluated by confocal microscopy (Figure 2E), as Hsp90 is the target protein of the prototype drug (CTPRAu) that would be encapsulated in mKate2 EV as part of this study. These results demonstrate the ability of 10K-EVs and 100K-EVs, derived from basal mKate2 NIH-3T3 fibroblasts, to be internalized in TGFβ-activated fibroblasts.

### Encapsulation of a new generation antifibrotic drug in 10K-EVs and 100K-EVs derived from mKate2 fibroblasts

**Figure 3:**
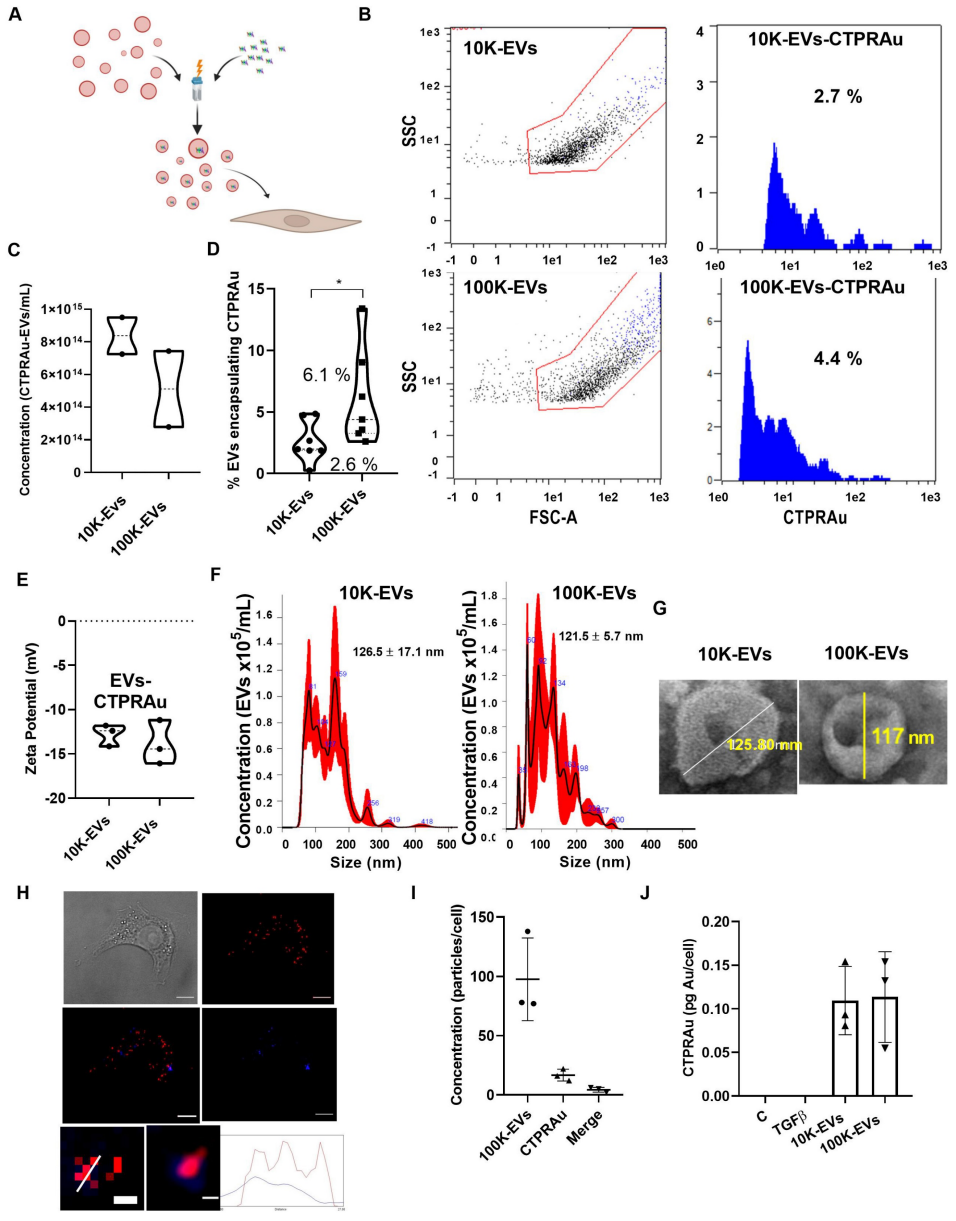
** Internalization of mKate2 10K-EVs-CTPRAu and mKate2 100K-EVs-CTPRAu in NIH-3T3 cells. A**) Cartoon depicting the encapsulation of CTPRAu in m-Kate EVs and its delivery to TGFβ-activated NIH-3T3 cells. **B**) Flow cytometry analysis of a representative assay showing the percentage encapsulation of CTPRAu (blue spots and blue area under the curve) in mKate2 10K-EVs (2.7%) and mKate2 100K-EVs (4.4%) after electroporation. **C**) Violin plot indicating the concentration of EVs (nº of EVs/mL) obtained after encapsulation of CTPRAu. **D**) Violin plot showing significant differences (*p < 0.05, Mann Whitney test) in percent encapsulation of CTPRAu in mKate2 10K-EVs compared to mKate2 100K-EVs. The results shown are the mean value of 7 independent flow cytometry assays.** E**) Charge measurements at a distance from the particle (zeta potential) in mV in both types of EVs after CTPRAu encapsulation, mKate2 10K-EVs-CTPRAu and mKate2 100K-EVs-CTPRAu; n = 3 independent experiments.** F)** NTA measurements of the concentration and distribution of populations in both types of EVs after CTPRAu encapsulation, mKate2 10K-EVs-CTPRAu (left panel) and mKate2 100K-EVs-CTPRAu (right panel); in red the standard deviation of the mean value; n = 4 independent samples measured.** G**) Representative electron microscopy images of mKate2 10K-EVs-CTPRAu and mKate2 100K-EVs-CTPRAu. The lines indicate the diameter of the EVs in nm.** H)** Four panels depicting a TGFβ-activated (1 ng/mL) NIH-3T3 cell treated with mKate2 100K-EV-CTPRAu: - brightfield; - confocal microscopy image of mKate2 100K-EVs (red fluorescence); - confocal microscopy image of CTPRAu (blue fluorescence); - merge image of the red and blue fluorescence confocal images. Scale bar: 10 μm. The bottom two panels show a detail representative detail of mKate2 100K- EV-CTPRAu in activated-NIH-3T3 cells pixelated and unpixelated where the red/blue fluorescent signals are merged indicating detection of a mKate2 100K-EV that has encapsulated CTPRAu. Scale bar: 40 nm. Bottom graph showing the variation of fluorescence intensities (red and blue) along the diameter line of the mKate2 100K-EV-CTPRAu.** I**) Quantification of red and blue fluorescent spots to show the number of mKate2 100K-EVs, mKate2 100K-EV-CTPRAu and CTPRAu molecules detected in NIH-3T3 cells activated with TGFβ (1 ng/mL) and treated with mKate2 100K-EV-CTPRAu. ImageJ software used for quantification (n = 3). **J**) Quantity of Au detected in mKate2 10K-EVs-CTPRAu and mKate2 100K-EVs-CTPRAu by ICP-MS; results shown are the mean value of 3 independent assays.

Therapeutic molecules, including siRNA, miRNA, mRNA, protein-based drugs and chemical drugs can be encapsulated in EVs 32-35. Encapsulation efficiency is a major challenge of cell-based encapsulating methods, where cargo is delivered into donor cells by cell endocytosis 36, and non-cell-based encapsulating methods, including direct electroporation, sonication, incubation, and transfection of EVs 37. Despite, the low amount of therapeutics present in EVs, EVs display similar therapeutic effects to those achieved by conventional drug delivery methods 38. We aim to investigate whether 10K-EVs and 100K-EVs secreted by mKate2 NIH-3T3 fibroblasts would enable the loading of therapeutic molecules and be able to be exploited as biologic drug nanocarriers. For this purpose, we employed the non-cellular encapsulation method of electroporation for the prototype drug CTPRAu (therapeutic protein-Au nanomaterial hybrid) previously tested by our group as an antifibrotic drug 25 (Figure 3A). 1 mM of CTPRAu in PBS was electroporated into EVs (10.0x10^14^ EV/mL) by 3 pulses of 400 mV. The experimental set up and fluorimetric detection of EVs, after washing away the supernatant containing the unencapsulated CTPRAu, were evaluated and results are shown in Figure S3A-E. Figure 3B shows the analysis of the number of CTPRAu containing-EVs from the total EVs population (blue spots) using Macs protocol adapted to detect EVs by cytometry. The area under the curve (AUC) of Figure 3B indicates the percentage of CTPRAu loaded in the EVs of a representative assay. After encapsulation, the total EV concentration was of 8.4x10^14^ ± 1.6x10^10^ 10K EVs/mL and 5.1x10^14^ ± 3.3x10^10^ 100K EVs/mL (Figure 3C). From the total population shown in Figure 3C, we observed a significant higher percentage of encapsulation in the 100K-EVs (6.1% ± 3.9%) compared to the 10K-EVs (2.6 ± 1.7%) fraction (*p < 0.05) in seven independent encapsulation assays analyzed by flow cytometry (Figure 3D).

To determine if the loading of the EVs with the negatively charged CTPRAu (Z-potential = -25.25 ± 8.173 mV, and hydrodynamic diameter of 4.2 ± 0.5 nm) (Figure S3F-G) affects the previously calculated EV charge (-5.8 ± 1.2 mV for the mKate2 10K-EVs, and -8.4 ± 0.7 mV for the mKate2 100K-EVs) (Figure 1F). We analyzed the Z-potential of mKate2 10K-EV-CTPRAu and mKate2 100K-EV-CTPRAu. Values of -12.8 ± 1.2 mV, and -13.9 ± 2.5 mV for 10K-EVs and 100K-EVs, respectively, was observed (Figure 3E), indicating that CTPRAu loading into EVs reduced their Z-potential almost two-fold in both types of EVs compared to EVs devoid of CTPRAu (*p < 0.05).

As in Figure 1 C-D, we measured the diameter of the total population of EVs including the 2.6 ± 1.7% of 10K EVs and the 6.1% ± 3.9% of 100K-EVs that contained CTPRAu. The mean diameter of 10K-EVs and 100K-EVs populations were 126.6 ± 17.1 nm and 121.5 ± 5.7 nm, with the largest populations measuring 159.0 ± 2.2 nm and 92.0 ± 1.4 nm, respectively (Figure 3F). Transmission Electron Microscopy (TEM) images of samples containing mKate2 10K-EVs-CTPRAu and mKate2 100K-EVs-CTPRAu showed that the morphology of these populations remained unchanged after electroporation and ultracentrifugation (Figure 3G).

To confirm the successful delivery of CTPRAu inside TGFβ-activated NIH-3T3 cells, by CTPRAu containing EVs, CTPRAu was detected by confocal microscopy through the fluorescence of the nanomaterial 25 and colocalization with EVs (mKate2 fluorescence) was determined (Figure 3H). To this end, 4x10^4^ ± 1x10^1^ TGFβ-activated NIH-3T3 cells were treated with 2.5x10^9^ ± 8.5x10^8^ EVs/mL mKate2 100K-EV-CTPRAu; the 100K-EV fraction was chosen because of its better encapsulation efficiency compared to 10K-EVs (Figure 3D). Figure 3H shows a representative example of mKate2 100K-EV-CTPRAu detection in TGFβ-activated NIH-3T3 cells, by confocal microscopy. As in Figure 2C-D, ImageJ software filters were utilized to quantify EVs and CTPRAu by their fluorescent signal intensity (Figure S4A-B). The lower panels of Figure 3H showed mKate2 100K-EV colocalizing with CTPRAu shown by detecting colocalization of both the fluorescent signals and their spectra. Figure S4C showed the analysis to detect the colocalization of EVs (red fluorescence) and CTPRAu (blue fluorescence) showing the spectra of both particles. We quantified the number of internalized mKate2 100K-EVs-CTPRAu in cells from 3 independent assays (Figure 3I). We detected similar internalization values for mKate2 100K-EVs-CTPRAu as for unloaded (CTPRAu void) mKate2 100K-EVs (97.7 ± 34.9 EVs/cell) (Figure 3I). 18.0 ± 13.9 CTPRAu/cell were visualized, of which 4.3 ± 2.1 CTPRAu/cell were co-localized with mKate2 100K-EVs (Figure 3I), 36 h after EV administration onto TGFβ-activated NIH-3T3 cells. To confirm the presence of CTPRAu inside active fibroblasts (10x10^6^ ± 1x10^3^) transported by mKate2 EVs, we measured the gold content per cell by ICP-MS. The results showed 0.11 ± 0.04 pg of Au/cell treated with 10K-EV and 0.11 ± 0.05 pg of Au/cell that were administered by 100K-EV (Figure 3J).

To test that 10K-EVs and 100K-EVs are not toxic nanocarriers, and in accordance with the MISEV2018 guidelines, which recommend to systematically report the level of cell viability in cultured cells producing or capturing EVs 5; we analyzed the cell number in both basal mKate2 NIH-3T3 fibroblasts secreting EVs and TGFβ-activated NIH-3T3 fibroblasts receiving EVs. In both cases, we observed cell proliferation during the assay with a three-fold increase in secreting cells during the 5 days of EVs secretion (Figure S4D). Importantly, the cell number was also unaffected by the administration of EVs or EVs containing the CTPRAu drug, with around 8x10^5^ cells per well in control, TGFβ-activated, or TGFβ-activated-treated with EVs at 36 h post EV administration (Figure S4E). These results showed that cell viability measurements did not reflect any decrease in normal proliferation rates after administration of mKate2 EVs-CTPRAu, nor did the secreting mKate2 EVs. Moreover, we previously demonstrated that direct administration of CTPRAu was not toxic to TGFβ-activated NIH-3T3 cells 26.

We showed that 10K-EVs and 100K-EVs secreted by mKate2 NIH-3T3 fibroblasts were uptaken by TGFβ-activated fibroblasts. Simultaneously, we observed mKate2 EVs transported CTPRAu to the target cells. Although electroporation of EVs altered the charge and size distribution of EVs, the diameters of the most abundant population in both the 10K-EV and 100K-EV fractions were unaffected. The changes observed in the nanocarriers did not alter their internalization capacity in profibrotic fibroblasts.

### Antifibrotic action of mKate2-EVs-CTPRAu

Collagen Ia1 (COL I), collagen IIIa1 (COL III), and fibronectin-1 (FN I) are known to be the main fibers overexpressed and accumulated in the extracellular matrix during stressful profibrotic events 39. Therefore, they can be utilized as specific molecular markers of fibrosis. Thus, the detection of mRNA expression levels of these molecules by qPCR can be a reliable assay to verify the cellular production of profibrotic markers after administration of exogenous particles. We aimed to determine whether administration of mKate2 10K-EVs-CTPRAu and mKate2 100K-EVs-CTPRAu could efficiently reduce fibrosis at the molecular level by measuring the mRNA expression of these three markers in TGFβ-activated receptor fibroblasts.

We separately measured the antifibrotic activity of mKate2 10K-EVs and mKate2100K-EVs in fibrotic fibroblasts. In addition, EVs alone have shown to have therapeutic action in different studies 40, therefore, we also analyzed the antifibrotic effect of unloaded-EVs. COL I, COL III, and FN I mRNA expression was measured in TGFβ-activated NIH-3T3 (4x10^4^ ± 1x10^1^) treated with CTPRAu loaded and unloaded mKate2 100K-EVs (7.0x10^9^ ± 4.0 x10^7^ EVs/mL) or CTPRAu loaded and unloaded mKate2 100K-EVs (2.5x10^9^ ± 8.5x10^7^ EVs/mL). Three assays with three independent qPCRs per condition and each condition measured in triplicate were used to assess the variation of COL I, COL III and FN I gene expression. Our previous research demonstrated that the reduced transcript levels of COL I, COL III, and FN I indicate down-regulation of the profibrotic signaling cascade in TGFβ-activated fibroblasts and therefore a reduction of pathogenic fiber accumulation in the extracellular matrix 16.

**Figure 4:**
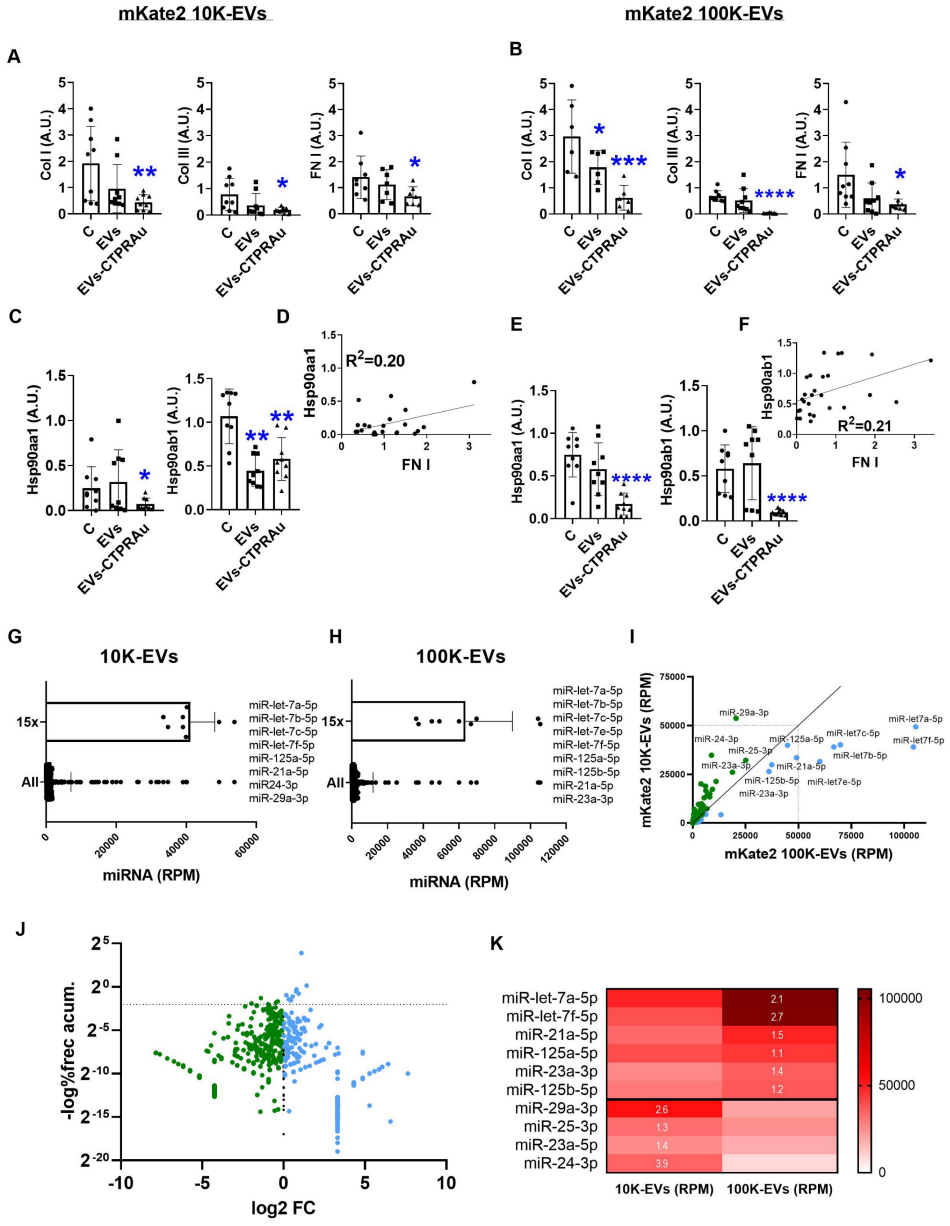
** Down-regulation of profibrotic markers after mKate2-CTPRAu EVs treatment of TGFβ-activated NIH-3T3 cells. Analysis of miRNA content of mKate2 EVs. A,B,C,E**) Relative gene expression of COL I, COL III, FN I, Hsp90aa1 and Hsp90ab1 36 h after treatment of TGFβ (1 ng/mL) activated NIH-3T3 (4x10^4^) with mKate2 EVs or mKate2 EVs-CTPRAu. (**A,C**) treatment with mKate2 10K-EV/mL or mKate2 10K-EVs-CTPRAu/mL (7.0x10^9^ ± 4.0 x10^9^) (**B,E**) treatment with mKate2 100K-EV/mL or mKate2 100K-EVs-CTPRAu/mL (2.5x10^9^ ± 8.5x1^8^). Significant variations were normalized with respect to the internal control (GAPDH gene). Experiments, n = 6-9 assays from n = 3 independent experiments. Mann-Whitney test: *p < .05, **p < .005, ***p < .0005, ****p < .0001; **D,F**) Linear regression and Pearson correlation analyses showing positive and significant correlation between Hsp90aa1 and FN-1 (Y = 0.1401 * X + 0.01194) (**D**); Hsp90ab1 and FN I (Y = 0.2026 * X + 0.543) (**F**) gene expression in TGFβ (1 ng/mL) activated NIH-3T3 cells and treated with mKate2 10K-EVs, or mKate2 10K-EVs CTPRAu. **G,H**) Bar graph with values of total microRNA (All) and values corresponding to miRNAs 15 times the mean (15x) in mKate2 10K-EVs (**G**) and mKate2 100K-EVs (**H**) and in reads per million (RPM). **I**) XY plot representing all miRNAs tested with a clear visualization of the highest RPM values obtained in mKate2 10K-EVs (green dots) and in mKate2 100K-EVs (blue dots). **J**) Volcano plot representing the distribution of miRNAs presence highlighting the most abundant and differentially concentrated in mKate2 10K-EVs (green dots) or mKate2 100K-EVs (right) with Y-axis showing the logarithm of the complement of the percentage associated with the cumulative frequency of RPM for each miRNA, and the X-axis showing the log2 of the fold change (FC). Above the dot line a similar distribution as in **I** panel can be observed. **K**) Heat map (in RPM) of the selected miRNAs from **G** and **H** (15x values) and corresponding to the miRNAs with more than 1.2-fold change (FC) abundance (white numbers inside red boxes indicate FC) in 100K-EVs vs 10K-EVs (upper values) or 10K-EVs vs 100K-EVs (lower values).

Figure 4A-B showed a significant reduction of COL I mRNA levels mediated by 10K-EVs-CTPRAu (**p < 0.005) and by 100K-EVs-CTPRAu (***p < 0.0005), COL III by 10K-EVs-CTPRAu (*p < 0.05) and by 100K-EVs-CTPRAu (****p < 0.0001) and of FN I (*p < 0.05) by both EV fractions, 36 h after EV addition to activated NIH-3T3 cells. We did not find an antifibrotic effect in cells treated with mKate2 10K-EVs (Figure 4A). In contrast, we observed an antifibrotic effect in cells treated with mKate2 100K-EVs (Figure 4B) shown by significant reduction of COL I (*p < 0.05). Moreover, we observed a greater downregulation of COL I (***p < 0.0005), and COL III (****p < 0.0001) in fibroblasts treated with mKate2 100K-EVs-CTPRAu (Figure 4B) than the reduction obtained for COLI (*p < 0.05) and COL III (**p < 0.005) in fibroblasts treated with 10K-EVs-CTPRAu (Figure 4A). The difference in the level of reduction by the two different EV fractions could be due to the higher percentage of mKate2 100K-EVs that successfully encapsulated CTPRAu (Figure 3B-D), a significant different efficacy of mKate2 100K-EVs over mKate2 10K-EVs as an antifibrotic treatment itself p < 0.05* (Figure 4B), or both.

As we previously reported, TGFβ-triggered fibrosis in NIH-3T3 by activating the expression of novel TGFβ signaling pathway molecules, including TGFβ's membrane receptors (TGFβRI and TGFβRII), and associated binding partners such as Hsp90 and key cytosolic secondary messengers. These molecules drive the profibrotic signal to the cell nucleus, ultimately leading to the expression and secretion of profibrotic molecules 41. Among them, Hsp90 has been consistently identified by our group and others as a protein involved in fibrosis progression 16, 42, 31 and has been defined as a target for decreasing fibrotic processes with gene expression modifications in fibrotic models 25. For this reason, we evaluated the expression of the cytosolic isoforms of Hsp90, Hsp90α stress inducible isoform and Hsp90β constitutively active isoform, in study (Figure 4C-E).

Previously, we described CTPRAu as an inhibitor of Hsp90 *in vitro* and *in vivo* 25-26. Accordingly, here we detected a significant reduction in Hsp90 transcript levels of both isoforms in TGFβ-activated fibroblasts treated with mKate2 10K-EVs-CTPRAu, and a highly significant reduction (****p < 0.0001) by mKate2 100K-EVs-CTPRAu, 36 h after administration of EVs on TGFβ-activated fibroblasts (Figure 4C-E). Notably, both isoforms of Hsp90 are largely reduced (****p < 0.0001) in the presence of 100K-EVs-CTPRAu but not when mKate2 100K-EV are not loaded with CTPRAu (Figure 4E). This result indicates that inhibition of Hsp90β by CTPRAu is affecting its mRNA expression. In contrast, the lower number of 10K-EVs encapsulating CTPRAu (2.6 ± 1.7%) could be behind the absence of significant differences in the reduction of Hsp90ab1 gene expression between mKate2 10K-EVs and mKate2 10K-EVs-CTPRAu treatments (Figure 4A).

To determine whether Hsp90α and/or Hsp90β could serve as new efficacy markers of an antifibrotic treatment using fibroblast-derived EVs, we performed correlations between the mRNA of classical markers studied, such as COL I, COL III and FN I, and the mRNA of both cytosolic isoforms of Hsp90 (Hsp90α and Hsp90β). To this end, gene expression values from the assays shown in Figures 4A-E were analyzed together to test for correlative events. Significant positive correlations were found between Hsp90α (Hsp90aa1 gene) and FN I gene expression in TGFβ-activated fibroblasts treated with mKate2 10K-EVs with and without CTPRAu encapsulation (R^2^ = 0.20) (Figure 4D). In the case of mKate2 100K-EVs with and without CTPRAu encapsulation correlation was found between Hsp90β (Hsp90ab1 gene) and FN I (R^2^ = 0.21) (Figure 4F). However, we could not find any correlation between the other profibrotic markers.

These results indicated that mKate2 EVs can efficiently transport CTPRAu into target cells to exert antifibrotic actions. Additionally, the correlation between the expression of Hsp90α and Hsp90β, and FN I suggested that these proteins have the potential to become biochemical markers of the antifibrotic effect of CTPRAu delivered by 10K-EVs and 100K-EVs.

### EVs miRNA content related to the antifibrotic action of mKate2 100K-EVs

EVs contain a variety of bioactive molecules, including proteins and nucleic acids (such as miRNAs) which contribute to cell function. MiRNAs are important components of EVs which mediate many of their functional effects. Thus, their detection offers key information regarding cell function regulation. We analyzed the miRNAs content of mKate2 100K-EVs and mKate2 10K-EVs. From the total 681 miRNA detected in mKate2 10K-EVs and the total 515 miRNAs detected in mKate2 100K-EVs, we found that 455 miRNAs were common in both type of EVs. 96 miRNAs were uniquely present in mKate2 10K-EVs and 130 were present only in mKate2 10K-EVs. These type-specific miRNAs were in very low number of reads suggesting a low likelihood of having a key role in the effect of the EVs on cell behavior (Figure S5). We detected a pool of 10 miRNAs (miR-let-7a-5p, miR-let-7b-5p, miR-let-7c-5p, miR-let-7e-5p, miR-let-7f-5p, miR-125a-5p, miR-125b-5p, miR-21a-5p, miR-23a-3p, miR-25-3p) in mKate2 100K-EVs; and a pool of 11 miRNA (miR-let-7a-5p, miR-let-7b-5p, miR-let-7c-5p, miR-let-7e-5p, miR-let-7f-5p, miR-125a-5p, miR-125b-5p, miR-21a-5p, miR24-3p, miR-25-3p, miR-29a-3p) in mKate2 10K-EVs as the most abundant (15x fold over the mean values of miRNA general content) (Figure 4G-H). Within these abundant miRNAs, Figure 4I-K highlighted miR-let-7a-5p and miR-let-7f-5p above 2-fold change in mKate2 100K-EVs vs mKate2 10K-EVs. In previous studies, treatments with miR-let-7a-5p were pointed as an efficient suppressor of fibrosis via reducing TGFβ/SMAD signaling pathway 43. The other highly abundant miRNA and differentially present in mKate2 100K-EVs compared to 10K-EVs was miRNA miR-let-7f-5p whose defined experimental target is TGFβ-R3 44. This is a membrane proteoglycan and TGFβ regulator that often functions as a co-receptor with the ability of modulate TGFβ receptors I and II, and its pro-fibrotic signaling cascade. Moreover, miRNA let-7f downregulates PI3K signaling pathway in fibrosis via targeting of PIK3 45. Regarding miRNAs with high abundance and 1.3-1.4 FC (Figure 4K) were miR-23a-3p that also participate in the antifibrotic process by attenuating the fibrosis response 46, and miR-21a-5p and miR125b-5p, which have not previously been described to have antifibrotic activity. The abundant and differential presence of these miRNAs could be part of the antifibrotic differences observed between mKate2 100K-EVs and mKate2 10K-EVs.

Below, we described the over-accumulated miRNA in mKate2 10K-EV compared to mKate2 100K-EV.

### *In vivo* detection of EVs and CTPR cargo in target organs

**Figure 5:**
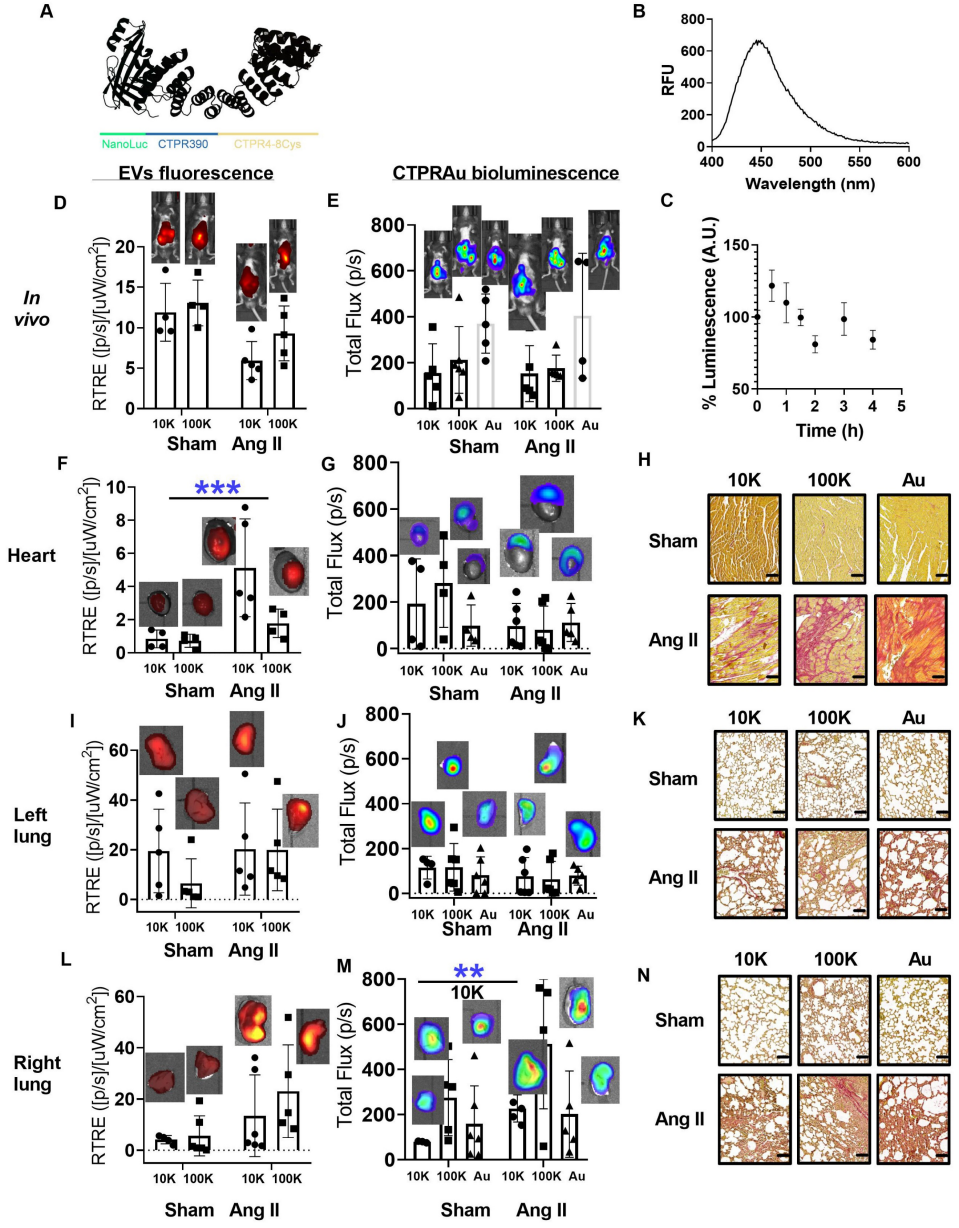
** Detection of mKate2 EVs and bCTPR/CTPRAu *in vivo* and* ex vivo* in target organs. A**) Schematic representation of the novel protein engineered by fusing NanoLuc with CTPR390 and a cysteine-based metal binding module to generate bCTPR. **B**) Luminescence spectrum of the generated chimeric bCTPR protein (3.8 μM) in the presence of substrate (FFz, 1 μM). **C**) Graph showing the stability of bCTPR luminescence (in mouse plasma at 37 °C) for 4 h. (**D,E,F,G,I,J,L,M**) Bar graphs with representative detections of *in vivo* (**D,E**) and *ex vivo* (**F,G,I,J,L,M**) EVs and bCTPR signals in Ang II group (fibrotic mice with Ang II minipump (1.5 mg/kg per day)) and sham group (healthy mice with saline minipump); n = 4 mice per group. Fluorescent signals were detected 2 h after intraperitoneal administration of 10K EV-bCTPR:CTPRAu/mL (10K) (6.8x10^11^ ± 1.6x1^9^) and 100K EVs-bCTPR:CTPRAu/mL (100K) (4.1x10^9^ ± 3.3x10^8^) to mice. Fluorescence of mKate2 EVs was detected in live mice, left ventricles of the heart, left lung, and right lung, respectively; n = 4 per group (**D,F,I,L**). Each point in the graphs corresponded to the relativized total radiant efficiency (RTRE) of the fluorescent signal of mKate2 EVs administered to mice (p/s)/(μWcm2). Each point in the graphs corresponded to the total flux in units (p/s) pertaining to the bioluminescent signal of bCTPR:CTPRAu (1:10) encapsulated in mKate2 EVs or freely administered. Bioluminescence of bCTPR:CTPRAu (1:10) was detected in live mice, left ventricles of the heart, left lung and right lung, respectively (**E,G,J,M**); n = 4 per group. Statistically significant variations correspond to **p < 0.005 Mann-Whitney test. (**H,K,N**) Picrosirius red staining showed fibrosis in longitudinal sections of the heart (**H**), left lung (**K**), and right lung (**N**) in all conditions studied (Sham and Ang II mice treated with mKate210K-EVs-bCTPR:CTPRAu, mKate2 100K-EVs-bCTPR:CTPRAu and CTPRAu. Scale bar: 100 μm.

As we previously demonstrated, *in vivo* fluorescent detection of drugs in animal models is an important tool to define the systemic behavior of new generation active antifibrotic molecules 16, 25. The previously reported CTPRAu consists of a protein-nanomaterial hybrid in which Au nanoclusters are stabilized by a metal binding domain based on a CTPR protein mutated to display several cysteines, which was fused the Hsp90-inhibiting module CTPR390. Herein, with the purpose of obtaining a bioluminescent CTPR (bCTPR) that would be compatible with the simultaneous detection of fluorescent EVs, we took advantage of the modularity, stability, and versatility of CTPR repeat proteins and fused CTPR390 and a metal-coordination domain similar to the one present in CTPRAu with NanoLuc luciferase (Figure 5A). NanoLuc was selected as bioluminescence source because it produces high intensity and glow-type luminescence. Additionally, it also presents excellent thermal and pH stability, conserving its activity at temperatures up to 55˚C and in a pH range between 5 and 7 47. The purification of bCTPR was confirmed by MALDI-TOF and SDS-PAGE electrophoresis (Figure S6A-B). Moreover, the CD spectrum of bCTPR exhibited the signature α-helical signal for CTPR proteins (Figure S6C), and thermal denaturation curves confirmed the outstanding thermal stability of the designed protein (Figure S6D-E). Figure 5B shows that the fusion of NanoLuc to CTPR did not affect its typical bioluminescence emission. The multifunctional bCTPR also showed good stability in biological media (mouse plasma) (Figure 5C) and *in vitro* (Figure S6F). The biocompatibility was studied by incubating bCTPR in mouse plasma at the same concentration used for *in vivo* assays, and it was verified that after 2 h, time point at which the bioluminescence is analyzed *in vivo,* the bCTPR kept around 81 ± 6% of the initial bioluminescence, maintaining its luminescence until the end of the assay (Figure 5C). In addition, the *in vitro* biocompatibility of the CTPR was evaluated in cell cultures. NIH-3T3 fibroblast cells were incubated with different concentrations of bioluminescent CTPR under standard cell culture conditions. Following 4 and 24 h of incubation, bCTPR did not present any evident cytotoxicity even at the highest concentration tested (Figure S6F).

To study the *in vivo* therapeutic effect of EV-encapsulated CTPRAu, the ability of the biotransporters to be imaged *in vivo*, and whether there was an improvement of the CTPRAu biodistribution, mKate2 EVs CTPRAu were administered to a fibrosis mouse model. To induce heart and lung fibrosis, mice were continuously infused with Ang II for 15 days through a subcutaneous osmotic mini-pump (minipump of Ang II (1.5 mg/kg per day). Sham mice were implanted with a minipump of saline solution. The hypertension promoted by Ang II generated mechanical damage in the heart and lungs as we shown in Figure 5K and Figure 5N. In addition to mechanical stress, Ang II activates pathological remodeling to end-organ damage in the short term. These reparative mechanisms, mainly fibrosis, in the long term, can lead to organ dysfunction, and in the worst-case scenario organ failure 48-49.

Herein, we used a procedure similar to the one recently described by our group 42 to detect fluorescence and bioluminescence signals *in vivo*. Figure S7 showed detailed description of the IVIS configuration procedure that allowed a clear signal from EVs and bCTPR. To define the most accurate excitation/emission setup, the fluorescence emission was assessed at different excitation wavelengths spanning from 660 nm to 780 nm and distinct emission wavelengths, 710 nm to 845 nm, followed by subtraction of the autofluorescence background (Figure S7A-B). *In vivo* (abdominal and thoracic areas) and *ex vivo* (selected organs) delimitations of Regions of Interest (ROI) are shown on Figure S7C-D.

Picrosirius red and Masson's trichrome staining were used to assess diffuse fibrosis in selected organs of Ang II-treated mice (Figure S7E). Figure S7F showed control and fibrotic heart and lungs staining for collagen in histologic preparations using Picrosirius red and Masson's trichrome. We selected the time point after intraperitoneal administration of mKate2 EVs at which *in vivo* fluorescent signal in the thoracic area was the highest. Figure S7G displayed the fluorescence intensity in the abdominal area (place of absorption) and thoracic area (place of interest where the heart and lungs are located) at different time points (t = 0 h, 30 min, 1 h, 2 h, and 2 h 30 min). We highlighted that the highest fluorescent intensity from EVs was achieved 2 h after EV administration and started decreasing 2 h 30 min after (Figure S7G). We previously demonstrated that a single intraperitoneal administration of free CTPRAu (1.0 mg/g mouse body weight), accumulated in fibrotic hearts of mice 7 days after the CTPRAu administration 25. The encapsulation of CTPRAu hybrids in EVs and its use as a biological therapeutic system aims to facilitate a faster accumulation of the drug in the target organs, compared to free CTPRAu administration. The presence of bioluminescent bCTPR and fluorescent EVs was evaluated* in vivo* in Sham (healthy) and Angiotensin II (Ang II) (fibrotic) mice. Bioluminescent CTPR was mixed with CTPRAu (bCTPR:CTPRAu) in a 1:10 ratio and encapsulated in mKate2 10K-EVs and mKate2 100K-EVs or administered free (non-encapsulated). CTPRAu and bCTPR are built on a similar protein scaffold that enables bioluminescence detection of EVs cargo without changing its structure. Using 1:10 bCTPR: CTPRAu ratio enables the detection of the system *in vivo* fluorescently (EVs) and bioluminescently (bCTPR:CTPRAu). Thus, the combination of bCTPR with CTPRAu allowed simultaneous bioluminescent tracking of the therapeutic cargo and fluorescent tracking of the EVs. *In vivo* and *ex vivo* colocalization of EVs and the encapsulated drug was visualized by superimposing fluorescence images of EVs and bioluminescence images of bCTPR in Figure 5D-M. We evaluated the fibrosis generated in the heart (Figure 5H) and lungs (Figure 5K-N) of Ang II mice, and showed the healthy heart and lungs of Sham mice as control tissues. Note that 2 h after treatment with EV-bCTPR:CTPRAu no significant reduction in fibrosis was observed (Figure 5H-N and Figure S7F). This is in agreement with previous studies showing evident antifibrotic effects one week after administration of CTPRAu, with no significant reduction in fibrosis 3 h after CTPRAu administration 25.

**Figure 6:**
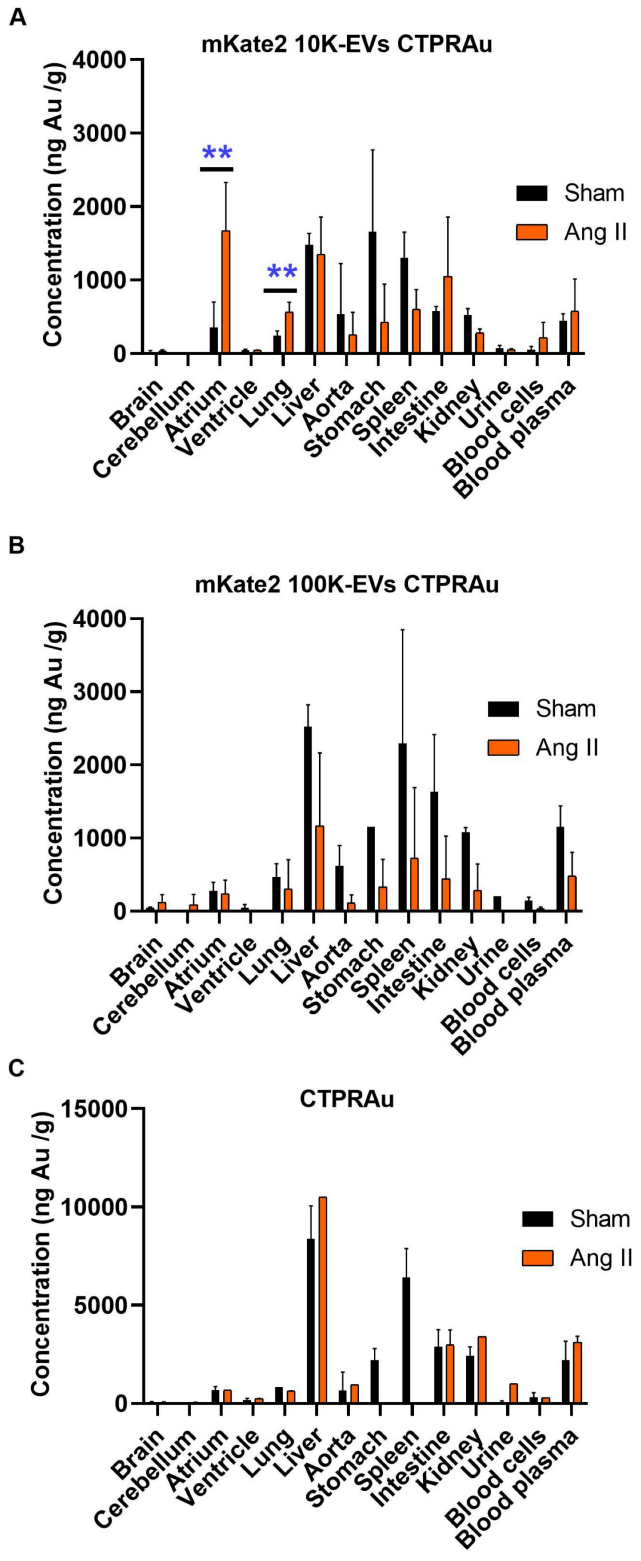
***In vivo* biodistribution of bCTPR/CTPRAu drug.** Au concentration in mice tissues (ng Au/g of tissue) were detected by ICP-MS in samples obtained 2 h after administration of bCTPR/CTPRAu (**A**) Drug encapsulated in mKate2 10K-EVs, (**B**) Drug encapsulated in mKate2 100K-EVs, or (**C**) Drug administered free to healthy (Sham) and fibrotic (Ang II) mice. Data with standard errors from n = 3 measurements of each tissue belonging to all animals studied in Figure 5. Statistically significant variations correspond to **p < 0.005 Mann-Whitney test.

To verify whether the encapsulation of CTPRAu in EVs could improve the previously observed low accumulation of CTPRAu in highly irrigated organs such as the heart and lungs 25, we compared the *ex vivo* signal intensity of the EVs-encapsulated, and free bCTPR:CTPRAu in these organs. *Ex vivo* measurements revealed that fibrotic mice showed significant accumulation of large EVs in the heart (***p < 0.0005) (Figure 5F). Interestingly, the drug encapsulated within mKate2 10K-EV appeared to accumulate in the upper zone of the heart, corresponding to the atria, giving no significant values when analyzing the total flow of the whole heart but offering significant accumulation of the drug transported in the analysis of the atrium separately (Figure 6A). In addition, we detected accumulation of bCTPR when transported by mKate2 10K-EVs in the right lung (**p < 0.005) (Figure 5M) but not in the left lung (Figure 5J). The mouse right lung has four lobes, and the left lung has one lobe. This difference in the number of lobules may be responsible for the observed differences in terms of cargo accumulation between right and left lungs at this specific time-point. An important observation from these assays is the ability of mKate2 10K-EVs to act as a nanotransporter of the drug to the destination in a short time (2 h) with a significant accumulation (**p < 0.005) of the antifibrotic drug in the fibrotic right lung and accumulation of the mKate2 10K-EVs in the heart. However, it is worth noting that no significant increase in the fluorescent signal from mKate2 10K EVs or in the bioluminescence signal from the bCTPR was observed in the fibrotic left lung.

### EVs miRNA content related to the increased targeting action of mKate2 10K-EVs

Within the abundant and differentially increased miRNAs detected in mKate2 10K-EVs vs mKate2 100K-EVs (Figure 4K), miR-24-3p was the most differentially present with a 3.9-fold in mKate2 10K-EVs compared to mKate2 100K-EVs. It has been described that *in vivo*, EVs containing miR-24 reached damaged hearts with infarct areas improving heart function. Therefore, transferring miR-24 in a paracrine manner through EVs reached the heart providing protective effects against damaged myocardium 50. The capability of targeting the cardiac endothelium by miR-24 in myocardial infarction has also been previously reported 51. We observed that fibrotic lung also accumulated mKate2 10K-EVs. Smad2 is a direct target of miR-24. Smad2 becomes a participant in transforming growth factor-β/Smad signaling fibrotic events in skeletal muscle fibrosis. Skeletal muscle fibrosis is a common feature in lung fibrosis 52. Another miRNA over 2-fold in mKate2 10K-EVs vs. mKate2 100K-EVs is miR-29a-3p (Figure 4K). Since miR-29a-3p has been described as a mayor regulator of genes associated with lung and heart fibrosis 53-54, it would not be surprising that it would target these organs. The other two miRNAs with FC over 1.3 were miR-25-3p (1.3 FC) suggested as a promising molecule for heart regeneration whose target is related to cardiomyocyte proliferation in the heart 55, and lung 56; and miR-23a-5p (1.4 FC) also associated with proliferation and overexpressed in damaged lungs 57 and targeting PI3K/AKT signaling pathway in the heart 58. The involvement in regulatory mechanisms related to the heart and lung of these miRNAs could help mKate2 10K-EVs to target these organs. From the rest of the miRNAs, 96 were detected only in mKate2 10K-EV (Figure S5) but the low number of reads detected would not indicate a key role in modifying cell behavior.

### Biodistribution of the drug CTPRAu after administration free or encapsulated in EVs

Current *in vivo* biodistribution results of EVs in mice are extremely heterogeneous depending on various factors such as EV doses, routes of administration, time points analyzed, tracking methods, EV isolation techniques, and EV size range 59. Therefore, it is crucial to provide robust biodistribution data. In this study, we performed unequivocal detection of the drug loaded in EVs, taking advantage of the presence of the non-biological metal gold in the CTPRAu. We quantified the amount of Au in different organs 2 h after intraperitoneal injection by ICP-MS (Figure 6). We verified that the *ex vivo* fluorescent signal detected in the heart and lungs belonged to EVs carrying drug (Figure 5D, Figure 5F, Figure 5I and Figure 5L) and that the *ex vivo* bioluminescent signal belonged to drug detection (Figure 5E, Figure 5G, Figure 5J, and Figure 5M) by quantifying the gold of the drug loaded in the EVs that reached the heart and lungs of all mice studied. We also provided information on drug accumulation in other organs such as brain, cerebellum, liver, aorta, stomach, spleen, intestine, kidney, blood cells, and internal media such as plasma and urine, of healthy and Ang II-treated mice (Figure 6).

We confirmed that both mKate2 10K-EVs and mKate2 100K-EVs were able to transport the drug encapsulated in them to different organs in healthy and fibrotic mice. Although the biodistribution patterns of drug transported in 10K-EVs and 100K-EVs are similar in healthy mice, *in vitro* results showed that 100K-EVs are better nanocarriers with higher antifibrotic efficiency. However, the transport of mKate2 10K-EVs is more efficient at achieving early accumulation in the atria and lung. Figure 6A-B showed the increased potential of fibroblast-derived mKate2 10K-EVs compared to mKate2 100K-EVs to reach and accumulate CTPRAu in the atria and lung compared to healthy controls (**p < 0.005). A comparison of the cargo in terms of miRNA contents revealed a significantly higher presence of miR-24 in mKate2 10K-EVs. Being miR-24 direct target (Smad2), overexpressed in fibrotic heart and lung, could be one of the explanations for the difference in biodistribution patterns. Related to the protein content, we observed Hsp90 significantly augmented in mKate2 10K-EVs compared to mKate2 100K-EVs. Numerous client proteins of Hsp90 have been identified in known cardiac and lung disease pathways, including MAPK signaling, PI3K/AKT (PKB)/mTOR, and TNF-α signaling 60.

We obtained similar results to those of a few studies investigating the biodistribution of large EVs administrated by intravenous injection, showing that EVs rapidly localized to lungs, liver, spleen and kidneys 61. Here, we demonstrated the presence of EVs in the same organs and in addition, were detected in the stomach and intestine, probably due to the intraperitoneal route of administration. Notably, the drug carried by 10K-EVs (Figure 6A) but not by 100K-EVs (Figure 6B) or freely administrated (Figure 6C) was present in significant amounts in the heart and lung of fibrotic mice 2 h after administration. Moreover, CTPRAu transported by 100K-EVs (Figure 6B) or freely administrated was detected mainly in the liver in both control and fibrotic mice.

## Discussion

In the present work, we have developed a biological drug delivery system based on trackable extracellular vesicles carrying within them a new generation antifibrotic drug. 100K fractions of fibroblast derived EVs were shown to have innate therapeutic activity on fibrosis models *in vitro*. Most significantly, 10K and 100K-EV fractions were able to effectively transport the antifibrotic drug to the disease tissue *in vivo*, thus demonstrating their biological carrier ability. In particular, NIH-3T3 fibroblasts were genetically manipulated to obtain fluorescently labeled EVs, which were further characterized *in vitro* and *in vivo* in terms of their cell internalization, accumulation in organs, as well as their antifibrotic activity once therapeutic drugs were encapsulated in these EVs. Fluorescent labeling of EVs allowed for ease of pharmacokinetic analysis in recipient cells 6, as well as in animal models, including the measure of their biodistribution, a critical characteristic for their potential clinical application 62. Fluorescently label EVs can be successfully produced by genetic manipulating their cell of origin to express fluorescent proteins fused to membranes targeting peptides that would result in the membrane targeted localization of the fluorescent tag 63. Alterations of the EV membrane can impact their physicochemical properties, surface protein profiles, and cellular uptake functions 64. In this study, we demonstrated that the encapsulation of a protein-based drug into EVs by electroporation also changed the size, and Z-potential of EVs, but their transport capability was unaffected.

Healthy fibroblast-derived EVs, with a key role in the organization of the extracellular matrix, act as active messengers in higher-order biological functions in pathological tissues 65. In terms of their biological characteristics, mKate2 NIH-3T3 EVs maintained paracrine behavior that allowed effective cell-cell communication and, in some cases, promoted essential physiological functions, as previously reported by others 65. This claim is supported by the observed down-regulation of collagen in profibrotic NIH-3T3 cell model treated with mKate2 100K-EVs containing antifibrotic miRNAs.

EVs modifications have been successfully employed to improve their therapeutic characteristics 34. In this way, the inherent therapeutic action of NIH-3T3 cell-derived 100K-EVs is linked to their carrier function, leading to an antifibrotic therapeutic action that is enhanced by the encapsulation of an antifibrotic drug, CTPRAu within the EVs. As such, the intrinsic antifibrotic action of mKate2 100K-EVs *in vitro*, including increased deregulation of a key profibrotic marker such as COL I and extending its effect to other important profibrotic fibers such as COL III and FN I, was further enhanced by encapsulating of CTPRAu into the mKate2 100K-EVs. The drug encapsulated mKate2 100K-EVs displays better *in vitro* antifibrotic activity than CTPRAu encapsulated in mKate2 10K-EVs. MiRNAs are key modulators of complex biological processes modifying cell function 43-46. The increased abundance of antifibrotic miRNAs in mKate2 100K-EVs (miR-let-7a-5p and miR-let-7f-5p) or the reduced presence of profibrotic proteins such as Hsp90, compared to mKate2 10K-EVs demonstrated a multifactorial antifibrotic action. The higher ability of mKate2 100K-EVs to internalize CTPRAu drug (6.1% ± 3.9% of encapsulated drug for mKate2 100K-EVs vs. 2.6 ± 1.7% for mKate2 10K-EVs), better cellular internalization features of mKate2 100K-EVs (94.7 ± 22.2 mKate2 100K-EVs/cell vs. 50.5 ± 32.5 for mKate2 10K-EVs/cell), and/or the intrinsic antifibrotic behavior observed for mKate2 100K-EVs could explain the better antifibrotic activity of mKate2 100K-EVs compared to mKate2 10K-EVs *in vitro*. The encapsulation of the CTPRAu antifibrotic drug into EVs showcased the biological magnification of its therapeutic activity. This concept highlights the potential clinical applications of new-generation therapies, where the biological carrier itself contributes to the therapeutic activity of the drug. 66.

The presence of fluorescent markers indicated that target cells were incorporating the purified EVs. It is a challenge to develop models that can accurately predict drug delivery efficacy during treatment. As previously described in other mammalian cells 67, mKate2 EVs were visibly internalized by TGFβ-activated fibroblasts, and antifibrotic effect was observed, as measured by the reduction of COL I, COL III and FN I expression in target cells. In addition, we have provided a new potential marker that can be used to assess the effectiveness of EV-mediated antifibrotic activity.

The key regulator of proteostasis, Hsp90, involved in the correct folding of numerous proteins in eukaryotic cells 68, was shown to be a valuable profibrotic marker in various diseases 16, 42, 69. We observed significant positive correlations between Hsp90 and FN I in both control and EV-treated activated fibroblasts. Fibronectin fibers are essential for the proper formation of the extracellular matrix, as they contribute to collagen deposition. They also are a key contributor of increased proliferation of profibrotic fibroblasts in heart-related 70 and lung-related 71 diseases, among others.

Interestingly, we found Hsp90 isoform-specific expression correlated with FN I expression, depend on the type of mKate2 EVs fractions used. Hsp90α (the stress-inducible Hsp90 isoform) levels correlated with FN I in cells treated with mKate2 10K-EVs, whereas Hsp90β (the constitutively active Hsp90 isoform) expression levels correlated with FN I levels in cells treated with mKate2 100K-EVs. Although both isoforms possess identical functional activities in chaperone complexes, Hsp90α are often overexpressed and secreted during cellular stress events 72, and they exhibit significantly different behavior with respect to substrate interactions under stress conditions 73. One interesting link, is the fact that exogenous extracellular Hsp90 has been shown to increase incorporation of FN fragments into fibrils within the ECM, suggesting that Hsp90 may regulate FN matrix assembly through its interaction with N-terminal FN domains 74. These observations merit further investigation to understand how the expression of Hsp90 isoforms would be crucial in evaluating diseases progression. The basic principle behind the future of personalized medicine is the targeted therapy administration and controlled release of therapeutic drugs. This study aimed to validate a biological method of administrating the therapeutic drug directly to the damaged organ or area, while minimizing the exposure of the rest of the body to the free drug. In this manner, therapeutic doses can be achieved at lower drug concentrations, avoiding side effects due to multi-target interactions, high drug doses, the associated toxicities, and the unnecessary exposure of healthy tissue to the therapeutic drug 75. For a drug to effectively reach its intended target and positively affect the diseased tissue, it is desirable to design therapeutic systems that interact with a specific organ, cell, or group of cells. The drug carrier must be specially designed for efficient transport of the drug to the pre-selected diseased sites 76. Ideally, a transporter-drug system is expected to be non-toxic, non-immunogenic, biochemically inert, biodegradable, biocompatible, and physico-chemically stable *in vivo* and *in vitro*. Furthermore, within the therapeutic system, the transporter should present a predictable and controllable pattern of drug release that closely mimics the endogenous release of molecules, including the characteristic of being easily and rapidly eliminated from the organism. In this way, the side effects of drugs, which are the main concern in most pharmacological interventions, could be mitigated. One possibility would be encapsulating drugs in effective selective biological nanocarriers, such as EVs 77. Non-cell-based EV loading methods, also known as exogenous or direct loading, deposits a therapeutic cargo into EVs after isolation. The *in vivo* efficiency of encapsulated treatments depends on the percentage of encapsulation. Insertion of large amounts of drugs into EVs is still technically challenging 78. In this study, we achieved low encapsulation rates of CTPRAu. Despite the low rates of encapsulation, the amount of CTPRAu present in EVs were still able to reduce fibrosis *in vitro* and successfully, reach and accumulate in target tissue, specifically the fibrotic atria of the heart and fibrotic lungs* in vivo*. Delivery of mKate2 EVs to the heart and lung occurred 2 hours after EV-administration. We hypothesize that distribution of the EVs to these organs is due, in part, to the high irrigation of the heart and lungs as compared to other organs and is less likely due to a specific target molecule that directs these EVs to the heart and lungs.

We describe the successful use of a versatile EV-based drug delivery system that enables i*n vitro* reduction of fibrosis and *in vivo* detection of the nanocarrier and its encapsulated drug to fibrotic target organs. The precise targeting of EVs to the desired cell type or organ of interest remains a significant obstacle in the successful translation of EVs-based drug delivery systems, in addition to the of off-target drug biodistribution limitations. *In vivo* efficacy was obtained once the drug-containing EVs reached relevant organs such as lungs and heart. MicroRNA, proteins and the rest of the active molecules present in mKate2 10K-EVs could be behind the mechanism for obtaining a better targeting with this population of EVs, as we envisioned. Regardless of the route of administration and cell source, most systemically injected EVs are rapidly taken up by macrophages in the reticuloendothelial system and eliminated. Depending on the cell source, they could modulate macrophage response, either to promote pro-resolving macrophage phenotype that supports tissue repair 79, as occurs with EVs from cardiosphere-derived cells in myocardial infarction 80; or to cause little or no effect, as occurs with mesenchymal stem cells-derived EVs in myocardial infarction 81, leading to EVs elimination. Drug delivery problems are largely limited to the pre-systemic distribution to the liver 82 and other elimination related organs, such as spleen and kidney, and could be addressed in preclinical assays by the administration of EVs. Time-dependent changes in EVs accumulation have been observed, consistently showing a significant presence of EVs in the liver shortly after administration 59. Here we compared the administration of the free drug and its encapsulation in 10K-EVs and 100K-EVs and obtained a better pharmacokinetic biodistribution in organs related to drug elimination, with a clear and significant reduction of drug accumulation in organs such as liver, kidney, and spleen. We also observed a lower accumulation in organs related to gastrointestinal tract absorption, such as the intestine and stomach, after intraperitoneal administration. After systemic administration, such as the intraperitoneal injection used in these studies, we and others 83 have demonstrated that 100K-EVs are rapidly (2 h) cleared from the blood circulation and accumulate in the spleen, liver, kidney, and gastrointestinal tract 59.

The detection of 10K-EVs exhibited a significantly higher presence in the fibrotic heart of mice compared to the healthy heart. The drug transported to the ventricles by 10K-EVs, did not show any difference in CTPR accumulation between healthy and fibrotic left ventricles. This is due to the significant accumulation of the drug in the atria of fibrotic hearts. The occurrence of CTPR accumulation in the atria of fibrotic hearts following the administration of the drug encapsulated in 10K-EVs did not occur after the administration of non-encapsulated CTPR. Previous studies conducted by our group have demonstrated that free CTPRAu can be detected in the hearts of both healthy and fibrotic mice 3 h after its administration. In those studies, we also demonstrated that a significant accumulation of the drug in fibrotic ventricles was observed only after waiting for 5 days following the administration of CTPRAu 25. We observed CTPR accumulation specifically in the fibrotic right lung when it is transported within 10K-EVs.

We purified fibroblast derived EVs taking into account that delivery of the EVs to the target cell/organ dependent on the selective uptake of EVs by recipient fibroblasts. It has been described that EV uptake occurs mainly when EVs and recipient cells share the appropriate ligand and receptor, which is more likely to occur when the donor cell, from which EVs are isolated from, and the EVs recipient cell, are the same cell type 84. However, there are also claims that EVs can be non-selectively incorporated into different types of cells 85. Regardless, we believe that the accumulation of CTPR in the atria and right lung at 2 h is a transient event that is likely to vary at shorter or longer time points after drug administration. We also consider that the presence of multiple lobes of the right lung of mice together with the intraperitoneal administration that might not ensure homogeneity in the distribution of substances, due to processes such as absorption and passage of membranes from the gastrointestinal tract to the circulatory system, might be some of the reasons behind the observed differences in drug accumulation between the right and left lungs.

It is well known that the *in vitro* efficiency of a new experimental therapeutic strategy can be impaired by poor *in vivo* biodistribution. This observation holds true for both mKate2 100K-EVs-CTPRAu and free CTPRAu, as studied at a 2 h time point after their administration. The expected biological effects of the experimental drug CTPRAu occurred mainly through internalization by recipient cells via endocytosis pathways, as we previously demonstrated 26, coincident with the mechanism of EVs uptake 84, 86. Thus, it appears that* in vivo* transport by mKate2 10K-EVs is better suited for transporting CTPRAu, resembling the natural vesicle-driven internalization detected in previous studies 26.

In conclusion, we have generated a biological trackable system based on EVs loaded with a new antifibrotic theranostic molecule with good antifibrotic functionality *in vitro*. The translation of this system to *in vivo* application already showed promising results with the accumulation of the transported drug in fibrotic heart and lung within the first 2 h after administration. This proof of concept allows for the possibility of using other drugs whose encapsulation may be applicable in the reduction of adverse effects, in addition to being a strategy that can be validated for this family of diseases.

## Materials and Methods

### Plasmid construction

pCDH-f_mKate2 vector including mKate2 into the lentiviral construct includes one of the most common fluorescent proteins, Red Fluorescent Protein attached to the N-terminus of the farnesyl group. Fluorescent farnesyl group incorporates into proteins such as c-Ha-ras, which allows their localization in cell membranes. (pCDH-f_mKate2 vector was a kind gift of Dr. N. Varela, IBBTEC-Universidad de Cantabria, Santander, Spain).

### Cell culture and mKate2 NIH-3T3 cell line generation

NIH-3T3 fibroblast cell line were purchased from American Type Culture Collections (Manassas, VA, USA). Cells were grown as monolayer at 37 ºC, under 5% CO2, in humidified incubators cultured in Dulbecco's modified Eagle's medium (DMEM Glutamax, Gibco), with 10% of Fetal bovine Serum (FBS, Gibco), 100 U/mL penicillin and 100 μg/mL streptomycin (Gibco). Cell in between passage 30-40 were assayed to produce membrane targeted and labeled mKate2 stable NIH-3T3 line. The new cell line was obtained by infection with lentiviral pCDH-f_mKate2 vector. Briefly, HEK293T cells were cultured in a 10 cm dish at approximately 20~30% confluence so that they did not reach confluence during the virus production process. 2 μg of pCDH-fmKate2 (plasmid of interest), 1.2 μg of psPAX2 (packaging plasmid) and 1.0 µg of pMD2G (VSVG envelope plasmid) were transfected together using Lipofectamine® LTX & PLUS™ reagent following the manufacturing recommended protocol. 24 h later, the medium was changed. After 24 h, HEK293T medium rich in viral particles was collected, filtered with a 0.45 µm filter and administered to the NIH3T3 culture at a 1:2 dilution in DMEN and with a final concentration of 8 µg/mL polybrene (sc-134220). This process was repeated two days in a row. After 48 h of incubation with viral particles, the medium was refreshed and antibiotic selection was initiated. MKate2 NIH-3T3 puromycin selected final cell line was checked for positive red fluorescence using epifluorescence microscopy. Regular NIH-3T3 regular cell line and mKate2 NIH-3T3 stable population are routinely tested using Myco detection Kit (Eurofins) for mycoplasma contamination. Only mycoplasma negative cells were used for experiments presented herein.

### EVs isolation

300,000 NIH-3T3 cells in passage 30-40 were plated per twenty-three 150 mm culture plates. Four days after, when 65% confluence was reached, the cells were washed twice with PBS and the medium was replaced with 14 mL of DMEM medium supplemented with 10% exosome-depleted FBS (Gibco A27208-01), 100 U/mL penicillin and 100 μg/mL streptomycin (Gibco). Five days after adding exosome depleted FBS medium, with cells at 80~90% confluence, we began the EVs isolation. Cells were counted and cell viability was determined using Trypan Blue (Invitrogen). Experiments were performed with cells showing viability ≥ 65%. To isolate EVs, conditioned media were collected and centrifuged at 2,000 g for 20 min at 4 °C to remove dead cells and debris. The resulting supernatant was centrifuged at 10,000 g for 90 min at 4 °C to obtain the 10K-EVs fraction. Subsequently, to obtain the 100K-EVs fraction, the supernatant was ultracentrifuged in 50 mL centrifuge tubes (3139-0050 Thermo Scientific™ Nalgene™) at 100,000 g for 90 min at 4 °C, using a Beckman Avanti J-30 ultracentrifuge. The 10K and 100K pellets were concentrated respectively using a 1.5 mL tube ultracentrifuge 082393315 OPTIMA MAX XP TABLETOP ULTRACENTRIFUGE. The 10K and 100K fractions were washed in 1 mL EVs-free PBS (by ultracentrifugation 100,000 g 90 min) and pelleted again in EVs-free PBS for further use.

### Western blot

40 µg of total protein from mKate2 10K-EVs and mKate2 100K-EVs (Nanodrop) were lysed using NuPAGE™ LDS buffer (Invitrogen™ NP0007). After boiling 5 min at 95 °C, samples were resolved on a 12% SDS-PAGE gel without reducing conditions. Resulting gels were then transferred to Nitrocellulose blotting membranes (Amersham™ Protan™ Premium 0.45 µm NC). The membranes were blocked with bovine serum albumin (Sigma-Aldrich A7906-50G) in TBS containing 0.1% Tween-20 (TBST) for 1 h at room temperature. After blocking, the membranes were incubated with the following primary antibodies diluted in 2% bovine serum albumin in TBS at 4 °C overnight: anti-CD-81 (1:150 dilution, monoclonal serum kindly gifted by Doctor Sanchez Madrid), anti-flotillin-1 (dilution 1:5000, sc-133153), anti-Syntenin-1 (dilution 1:500, sc-515538) anti-Hsp90 (dilution 1:1000, abcam ab13492). IRDye 800 CW and IRDye 680RD-conjugated secondary antibodies were used for immunodetection. The blots were visualized with the Odyssey® CLx Imager imaging system (LI-COR). Protein detection in the blots was quantified using ImageJ imaging software. Quantifications were normalized to total intensity.

### Flow cytometry

Flow cytometry measurements were performed using the MACSQuant® Analyzer 10 (#0130-096-343). Parameters were adjusted following the manufacturer's recommended protocol adapted for exosome population characterization: low flow rate (~10 µL/min), trigger on SSC at 4, all channels on hyper logarithmic scale, voltage set to 264 V for FSC and 219 V for SSC. EVs-free ultracentrifuged PBS (negative for Flotilin-1-1, Syntenin, and CD-81 assayed by WB) was used to evaluate buffer fluorescence background. MACSQuantify 2.11.1907.19925 software was used for analysis.

### Fluorescence Live Cell Imaging

4x10^5^ NIH3T3 cells were culture in 35 mm microscopy dishes (Ibidi µ-Dish 35 mm high Glass Bottom 81158). 36 h later, cells were washed and medium was replaced by Leibovitz microscopy medium (Gibco™ 21083027) and TGFβ (1 ng/mL). One minute of time laps was captured as baseline control. MKate2-10K or mKate2 100K-EVs were added to the cells. Cell images were recorded for up to 5 min.

Nikon eclipse Ti2 were used for live image acquisition with Orca flash 4 as a camera. A 60X oil objective was used with an opening of 1.4 mm. MKate2 (excitation: 588 nm, emission 633 nm) detection channel was mCherry channel using an exposure time of 100 ms, Led 575 nm 10% intensity excitation filter 578/21 nm (SPECTRA X lumencor) each 0.5 s. Differential interference contrast (DIC), with an exposure time of 30 ms, was taken every 3 pictures. After the time laps, cells were washed vigorously and fixed with 4% paraformaldehyde for further confocal microscopy.

### Immunochemistry of Hsp90αβ

Cells previously fixed were permeabilized for 30 min with 0.05% Triton-X in PBS. An overnight incubation at 4 ºC with anti-Hsp90 antibody (1:100 dilution, ab13492) was performed. The samples were washed with 0.05% Tween 20 in PBS, incubated for 1 h at room temperature with a secondary antibody (anti-mouse Cy5), washed in PBS. Fixed cells were stored in PBS at 4 ºC for subsequent confocal imaging.

### Confocal fluorescence microscopy

Leica SP5 confocal, 63X oil objective, 1.4 mm aperture and 2.5 zoom was used. Fluorescence of mKate2 EVs (excitation: 588 nm, emission 633 nm) was taken using mKate2 channel with 594 nm laser, 49% intensity, 610 nm excitation filter and 660 nm emission filter; CTPRAu (excitation: 390 nm, emission: 488 nm), DAPI channel, laser 405 nm, intensity 27%, excitation filter 415 nm, emission filter 500 nm; Cy5 from anti-Hsp90 immunofluorescence (excitation 633 nm, emission 647 nm), Far Red channel, laser 633 nm, intensity 43%, excitation filter 648 nm, emission filter 698 nm. Seven z-slices were acquired with a step size of 1.13 μm. LAS AF software was used to capture images.

At least three cells were captured from each condition to obtain representative images of the total number of EVs per cell. The images were analyzed using ImageJ software. A median filter was applied to each channel and a maximum intensity z-projection was performed for each channel separately. Once brightness and contrast were adjusted, the images were converted to binary to count the number of EVs in each cell using the find maxima tool. This tool determines the local maxima in an image and creates a binary image of the same size with particle per maximum. Analysis is performed on the existing selection of the cell analyzed. To select the EVs, Noise Tolerance is adjusted. Maxima are ignored if they do not stand out from the surroundings by more than this value. These adjustments set a threshold at the maximum value minus noise tolerance. The contiguous area around the maximum above the threshold is analyzed. For accepting a maximum, this area must not contain any point with a value higher than the maximum. Only one maximum within this area is accepted. Under these conditions the colocalization of mKate2 EVs and CTPRAu fluorescence was measured.

### Transmission Electron Microscopy

Negative staining was performed to be imaged by a JEOL- JEM 1011 transmission electron microscope (TEM) with a high-resolution Gatan digital camera. 3 μL of EVs in PBS were placed on a parafilm sheet so the formvar coated nickel grid was placed formvar side down on top of the sample for 5 min at room temperature. After that, grid was removed, blotted with filter paper, and placed into another drop of 0.5% uranyl acetate for 5 s, blotted again, washed two times with two drops of dionized water and air dried until observation. Transmission Electron Microscopy (TEM) images of mKate2 10K-EVs and mKate2 100K-EVs were recorded at an accelerating voltage of 80 kV and with magnifications ranging from 6000× to 29,000× using a GatanUltraScan 100 slow-scan CCD camera and software Gatan.

### Measurement of particle size distribution and particle number by Nanoparticle Tracking Analysis (NTA)

The particle size distribution and the number of particles in the collected EV suspension were measured using nano particle tracking analysis device (Malvern Panalytical NanoSight NS300 Instrument, UK). Briefly, EV suspensions were diluted in MilliQ distilled water (1:100) and loaded into measurement cassettes. The measurements have been carried out at room temperature, using 532 nm (green) laser source. The concentration and mean size were calculated for each sample based on three captures (each 60 s) and a total of 1489 frames. The calculations have been done via built-in software version NTA 3.4 Build 3.4.4.

### Surface potential of mKate2 10K-EVs and mKate2 100K-EVs

MKate2 EVs samples diluted in 1 mL of depleted PBS to reach a final concentration of 5x10^6^ particles/mL. Samples were added to the adapted cuvette for the Zeta Potential Analyzer (NANOTRAC WAVE II Microtrac analysis technology) during the preparation process. MKate2 EVs were measured thrice at 25 °C under the following settings: sensitivity of 85 a shutter value of 70, and a frame rate of 30 frames per s. ZetaView software was used to collect and analyze data.

### bCTPR design, cloning and molecular biology

CTPR390 and CTPR proteins were produced and characterized as described previously 87-90. CTPR390 was fused to CTPR4-8Cys by a block cloning strategy, resulting in CTPRAu 25. Then, to generate bCTPR, CTPR390-CTPR4-8Cys was fused to NanoLuc by isothermal assembly 91. The DNA encoding NanoLuc was kindly provided by Prof. Barbara Montanini group. The final DNA sequence, NanoLuc- CTPR390-CTPR4-8Cys, named bCTPR was cloned in a pET-HT vector and was confirmed by Sanger sequencing (Stab Vida).

### bCTPR expression and purification

Overnight cultures of *Escherichia coli* C41 (DE3) transformed either with NanoLuc-CTPR390-CTPR4-8Cys or CTPR8-24Cys-CTPR390 (bCTPR), were inoculated in a dilution of 1:100 in LB medium containing 0.1 mg/mL of ampicillin. Bacteria were grown under agitation at 37 °C until optical density of the culture was 0.6. Protein expression was then induced by the addition of 1 mM isopropyl β-d-thiogalactoside (IPTG). The bacterial cultures were then incubated overnight at 20 °C and finally centrifuged for 15 min at 4,500 rpm to obtain a cell pellet that was next resuspended in 50 mM Tris, 500 mM NaCl, pH 8.0 and frozen. The cell pellets were lysed by sonication and centrifuged for 1 h at 10,000 rpm. The soluble proteins were purified by affinity chromatography using a 5 mL HisTrap Q column (GE Healthcare). The His6-tag was then digested from the designed proteins by the Tobacco Etch Virus (TEV) overnight treatment at room temperature, and then purified once again by affinity chromatography (proteins of interest eluted in the flowthrough). The protein concentration was determined by measuring the absorbance at 280 nm, using extinction coefficients calculated from the amino acid composition of each protein. Purity and size of the proteins was confirmed by acrylamide electrophoresis gel (12%) imaged by GelDoc EZ Imager (BioRad), and by Matrix-Assisted Laser Desorption/Ionization-Time of Flight (MALDI-TOF). The sample preparation consisted of mixing 1 μL of sample with 2 μL of sinapinic acid matrix (in 50:50 water/acetonitrile with 0.01% TFA). The mass spectra were acquired by an Applied Biosystems Voyager Elite MALDI-TOF mass spectrometer with delayed extraction (Applied Biosystems, Framingham, MA, USA). This equipment is equipped with a pulsed N2 laser (λ = 337 nm) and it was used at an extraction voltage of 20 kV. The spectrum was obtained in positive reflection mode using delayed extraction with an average of 50-100 laser shots. The secondary structure was verified by circular dichroism using a Jasco J-815 (JASCO Corporation, Tokyo, Japan). CD spectra were obtained at 2.5 μM of protein using a cuvette with 1 mm path length. The thermal denaturation spectra were obtained by measuring the CD signal at 222 nm at the different temperatures with a gradient of 1 °C and measured after 5 s of the sample being at the target temperature.

### bCTPR bioluminescence

The bCTPR bioluminescence generation was evaluated using a BioTek Synergy H1 hybrid microplate reader after incubating 3.8 μM of bCTPR with 2 μM of its substrate, FFz in a final volume of 30 μL (phosphate-buffered saline, PBS, 20 mM Na2HPO4, 1.8 mM KH2PO4, 140 mM NaCl, 1.8 mM KCl, pH 7.4) in a black 384-well plate. The luminescence emission spectrum was recorded in RLU (Relative Light Unit) against wavelength (nm) plots through a monochromator (1 s of integration time), by top read, reading height of 7 mm, and gain of 100.

### Stability measurements of the bCTPR chimera protein

To mimic *in vivo* conditions, bCTPR was incubated with mice plasma at a final concentration of 3.8 μM. The protein and plasma mixture were incubated at 37 °C for 4 h under mild agitation. To measure the stability of bCTPR bioluminescence under these conditions, several samples of this mixture were withdrawn at the different time points (0, 0.5, 1, 1.5, 2, 3, and 4 h). Each sample was incubated with 1 μM of FFz and its bioluminescence measured under the same parameters mentioned above. The percentage of luminescence was calculated setting the bioluminescence signal of the sample time point 0 (right after adding the protein to the plasma) as 100%.

### *In vitro* cytotoxicity of bCTPR

To evaluate the cytotoxicity of bCTPR, NIH-3T3 fibroblasts at 80% confluence were treated with several concentrations of bCTPR for 4 and 24 h. After the treatment, the cells underwent 3 washes with DPBS. Subsequently, they were incubated with 0.1% resazurin (1 mg/mL in DPBS), which was diluted in DMEM medium at 37 °C and 5% CO_2_, for a duration of 3 h. The fluorescence intensity of resorufin (reduced resazurin) was then measured using a BioTek Synergy H1 hybrid microplate reader (λexc = 570 nm, λem = 600 nm), using a black well plate. The percentage of cell viability was calculated using non-treated/ control cells as 100% viability.

### Synthesis of protein-stabilized gold nanoclusters (CTPRAu)

CTPRAu were synthesized through biomineralization, by optimizing a protocol previously described 92. Initially the protein buffer was changed to 150 mM of NaCl, 50 mM phosphate buffer at pH 11.2 using a PD-10 desalting column. Then, 1 mL of protein at 10 μM was incubated with 48 equivalents of Au per protein (3.2 μL of HAuCl_4_ at 150 mM) at 50 °C for 72 h. After the synthesis, CTPRAu were centrifuged at 16,400 rpm for 1 h and subsequently concentrated using an Amicon filter with pore size of 10 kDa. Finally, the nanomaterials were purified using a PD-10 desalting column and the buffer changed to PBS for further storage and use.

### Electroporation of mKate2 10K-EV and mKate2 100K-EV for CTPRAu encapsulation

Once isolated, populations of mKate2 10K-EVs and mKate2 100K-EVs were resuspended in 500 µL of EV-free PBS and mixed with 500 µL of 1 mM CTPRAu. After 15 min shaking at room temperature, two electroporation pulses of 0.9-1.0 s were applied. The electroporation parameters set were 400 V, 125 µF. After electroporation, the EVs populations were isolated by consecutive centrifugations at 10,000 g and 100,000 g to get rid of unencapsulated CTPRAu.

### Generation of the bCTPR:CTPRAu mixture and its encapsulation into EVs for *in vivo* and *ex vivo* studies

To visualize *in vivo* (bioluminescence) and quantify *ex vivo* (Au detection) the CTPR390-CTPR4-8Cys protein structure loaded into EV, we generated the bCTPR:CTPRAu mixture in a ratio of 1:10. The bCTPR:CTPRAu cargo was created by mixing 300 µL of bCTPR (0.1 mM) and 500 µL of CTPRAu (1 mM). Next, populations of mKate2 10K-EVs or mKate2 100K-EVs resuspended in 200 µL of EV-free PBS were added to the bCTPR:CTPRAu mixture to obtain a final volume of 1 mL. The encapsulation protocol explained in the previous section was then applied. It should be noted that the *ex vivo* quantification by Au detection of the total CTPR390-CTPR4-8Cys present in the tissue measured 90% of the total CTPR390-CTPR4-8Cys molecules (bCTPR:CTPRAu, ratio 1:10).

### *In vitro* treatment with mKate2 10K-EVs and mKate2 100K-EVs void or loaded with CTPRAu

4x10^4^ NIH-3T3 acceptor cells were seeded in 12 well plates and incubated at 37 °C with 5% CO_2_. After 24 h, around 65% confluence was reached. At this point, 1 ng/mL of TGFβ and mKate2 10K-EVs or mKate2 100K-EVs at a final concentration of 7.0x10^9^ ± 4.0x10^9^ EVs/mL and 2.5x10^9^ ± 8.5x10^8^/mL respectively, were added to cells. 24 h and 36 h later, the medium was removed, and cells were stored at -80 °C for further experiments.

### Quantitative PCR analysis of cell samples

The total RNA from NIH-3T3 cells was obtained with TRIzol® reagent (Gibco BRL, Grand Island, NY). Complementary DNA was prepared from 0.5 μg total RNA by random priming using a first-strand cDNA synthesis kit (Promega Corp). Primers sequence for the Sybr green QPCR of COL Ia1, COL IIIaI, FN, Hsp90aa1, Hsp90ab1 were: COL Ia1 forward: TGGGGCAAGACAGTCATCGAATA; reverse: GGGTGGAGGGAG TTTACACG. COL IIIa1 forward: ACCCCATGATGTGTTTTGTGGCA; reverse: CAGGTCCTCGGAAGCCACTA. FN forward: CACCCACATGGCAGCTCACA; revers: ATGGGAACCCTGAAGCCAGC. Hsp90α forward: TTCCAGAAGATG AAGAGGAAAA; reverse: GTCACCAGTCGGTTTGACAC. Hsp90β forward: TCTACTTCATGGCTGGGTCA; reverse: AACTCGGGAAGAGCCTGAAT. The target mRNA expression levels were normalized to 14S levels, 14S forward: AGTGACTGGTGGGATGAAGG; reverse: CTTGGTCCTGTTTCCTCC TG. Relative quantification was expressed as fold-induction compared to control 14S in triplicate of three independent experiments.

### MicroRNA sequencing of mKate2 10K-EVs and mKate2 100K-EVs

Total small RNA from mKate2 10K-EVs (190 μL of 2.9x10^15^ EVs/mL) and mKate2 100K-EVs (190 μL of 1.9x10^14^ EVs/mL) was isolated with the isolation kit (Thermo fisher, ref.:4478545) following the specifications for enriching the sample in small RNAs. Final miRNA concentration obtained from mKate2 10K-EVs (4.4 ng/µL) and mKate2 100K-EVs (3.4 ng/mL) was measured with the Qubit miRNA assay kit (Invitrogen™ ref.: Q32880). Next Generation Sequencing of miRNA was performed by Macrogene Company (dna.macrogene.com) following this manufacturer's protocol. The construction of sequencing libraries by polyadenylation of the isolated RNA were used to provide a priming sequence for SMART smRNA Oligos 5´ and 3´. PrimeScript Reverse Transcriptase generated cDNA by adding non-templated nucleotides bound by the SMART smRNA Oligo-enhanced. Locked nucleic acid (LNA) technology was used for greater sensitivity. PCR amplification included full-length Illumina adapters. Resulting library cDNA molecules include sequences required for clustering on an Illumina flow cell. The libraries were validated by checking the size, purity, and concentration on the Agilent Bioanalyzer. The libraries were pooled in equimolar amounts and sequenced on an Illumina NovaSeq instrument. Image decomposition and quality value calculation were performed using the modules of the Illumina pipeline. Procedure for data analysis included: -Adapter trimming using cutadapt program 93. Trimmed reads, whose length is longer than 18 bp, and non-adapter reads were combined and regarded as processed reads for downstream analysis. -Clustering of the reads whose sequences and length are the same to minimize the sequence redundancy. -Ribosomal RNA filtering in order to eliminate rRNA. -Identification of known miRNA reads using miRDeep2 software algorithm to assign scores representing the probability that hairpins are true miRNA precursors 94. Normalization is performed using an R package called edgeR.

### Care of the mice

Live animal studies were approved by the University of Cantabria Institutional Laboratory Animal Care and Use Committee in compliance with the Guide for the Care 11 and Use of Laboratory Animals (ILAR, 1985) and were conducted in accordance with the “European Directive for the Protection of Vertebrate Animals Used for Experimental and Other Scientific Purposes” (European Communities Council Directive 86/606/EEC). Male C57BL6 mice between 16-20 weeks old used for this study, were housed in a 22 °C room with 12:12 h light/dark cycle and provided with food and water ad libitum.

### Subcutaneous implantation of osmotic minipumps and antifibrotic treatment administration

Random selection of 6 littermate mice was selected to generate the following groups under study.

- Sham group: Osmotic minipump implantation loaded with saline solution.
- Fibrotic group: Osmotic minipump implantation loaded with Ang II solution.
- Sham mKate2 100K-EVs CTPRAu treated group: Osmotic minipump implantation loaded with saline solution and intraperitoneal administration of 50 μL of mKate2 EVs encapsulating (100 μM) CTPRAu + bCTPR (1 μM) solution.
- Sham CTPRAu treated group: Osmotic minipump implantation loaded with saline solution and intraperitoneal administration of 50 μL of 1x10^6^ mKate2 EVs encapsulating CTPRAu (100 μM) + bCTPR (1 μM) solution.
- Fibrotic mKate2 100K-EVs treated group: Osmotic minipump implantation loaded with Ang II solution and intraperitoneal administration of 50 μL of 1x10^6^ mKate2 EVs encapsulating (100 μM) CTPRAu + bCTPR (1 μM) solution.
- Fibrotic treated group: Osmotic minipump implantation loaded with Ang II solution and intraperitoneal administration of 50 μL of 100 μM CTPRAu + bCTPR (1 μM) solution.

The osmotic minipumps containing Ang II or saline solution (sham) were implanted subcutaneously as follows. Mice were anesthetized with 2.5% isoflurane in 1.5 L/min O_2_ for the duration of the surgical implantation procedure. A mid-scapular incision is made, and a subcutaneous pocket is created for the micro-osmotic pump to be inserted. The minipump was previously filled with an aqueous solution of angiotensin II (0.70 mg) to infuse a dose of Ang II of 1.5 mg/kg per day with a time-regimen of 0.25 μL per h, 14 days, (Alzet) to the “Fibrotic” groups or saline to “sham” groups. The wound was closed with a suture. Mice were monitored daily for any discomfort and they were euthanized at the endpoint (1 week after surgery).

Under 2.5% isoflurane anesthesia in 1.5 L/min O_2_, mice were injected intraperitoneally with 100 μL of mKate2 EVs (6.8x10^11^ ± 1.6x10^9^ 10K EV-bCTPR:CTPRAu/mL and 4.1x10^9^ ± 3.3x10^8^ 100K EVs-bCTPR:CTPRAu/mL) or free bCTPR:CTPRAu at a concentration of 100 μM in a 1:10 bCTPR:CTPRAu ration (10 μM:90 μM). The percent encapsulation of bCTPR:CTPRAu in a 100 μM:1 mM ratio was 6.1% ± 3.9% for 100K-EVs and 2.6 ± 1.7% for 10K-EVs.

At the end of the study, organs and fluids were collected, flash frozen and stored at -80 °C until ICP-MS assays were performed. Blood was collected in heparinized tubes. To separate the plasma from the cellular content, a centrifugation for 10 min at 2,000 g 4 °C was carried out. Plasma and blood cells were stored at -80 °C.

### *In vivo* and *ex vivo* fluorescence imaging (IVIS)

Populations of mKate2 EVs described in the previous section were stained with the lipophilic fluorescent dye DiR (Invitrogen D12731) in order to visualize them with the *in vivo* imaging system (IVIS), as the excitation/emission wavelengths of mKate2 are not optimal for this equipment. We settled the *in vivo* region of interest (ROI) through oval circles delimiting the total signal detected with a long diameter of 5.68 cm and a short diameter of 3.44 cm in all mice studied. *In vivo* fluorescence and bioluminescence images were obtained with the Xenogen *in vivo* imaging system (IVIS, PerkinElmer). Mice were anesthetized with 2.5% isoflurane in 1.5 L/min O_2_ and were placed into the IVIS chamber.

Fluorescence images were obtained with excitation/emission filters 660/710 nm, 680/790 nm, 700/790 nm, 720/790 nm, 740/790 nm, 760/845 nm, 780/845 nm to generate the spectrum and perform spectral unmixing to remove the signal from tissue autofluorescence for visualization of DiR-stained EV populations both *in vivo* and *ex vivo*. The exposure time set in “auto-expose”. Adaptive FL Background subtraction correction and spectral unmixing were performed to eliminate auto fluorescence signal. *In vivo* bioluminescence from bCTPR was measured by acquiring total bioluminescence, exposure time “auto-expose”. To detect bioluminescence 50 μL of 0.22 µmol of Nano-Glo® *In Vivo* Substrate, FFz (Promega CS320501) was injected 15 min before imaging.

Following *in vivo* Imaging, mice were sacrificed and *ex vivo* fluorescence and bioluminescence photos were taken from heart and lungs. We set the *ex vivo* ROI through circles delimiting the total signal detected with a diameter of 1.76 cm. All organs were flash frozen and stored at -80 °C until ICP-MS assays were performed.

Acquired images were analyzed using the Live Image 4.7.2 software. Fluorescence from each mice and organ was quantified by relative total radiance efficiency RTRE [p/s] / [µW/cm²]. Bioluminescence was measured in total flux [p/s]. All measurements were normalized to each control.

### Histological analysis

Longitudinal heart and lung sections (4 µm) were obtained from treated mice (control and fibrotic). Organs were fixed in 4% paraformaldehyde, followed by gradient alcohol dehydration and paraffin embedding. COL fibers were stained with Masson's trichrome stain as well as Picrosirious red stain. Masson's trichrome staining was performed using Harrys' hematoxylin (Merk ref.:1.09253) and staining with Fuchina Ponceau (Acid fuchsine, merk ref.: 1.05231; and Xylidine Ponceau, sigma ref.: P2395) was used as contrast stain. Phosphomolybdic acid (Pancrea ref.: 1310031.1608) was used to label the collagen with light green (Merck ref.: 1.15941).

Picrosirious staining was performed by incubating Harrys' hematoxylin (Merk ref.:1.09253) as a contrast stain followed by Picrosirious red solution (SctTek ref.: SRS500) to stain the collagens. Complete scanning of all sections was performed in bright field with the Zeiss Axioscan Z1 scanner using the ×10/0.45 Plan Apo objective. Images were exported as JPG files with a pixel size of 0.884 mm/ pixel and analyzed with ImageJ 1.52i software.

### Inductively coupled plasma mass spectrometry (ICP-MS) of mKate2 EVs, cells and organs

Au (CTPRAu) concentration present in CTPRAu treated cells and mice was determined by ICP-MS. Briefly, NIH-3T3 pellet was obtained via centrifugation at 3,000 rpm for 10 min at room temperature of a trypsin detached and counted 10 cm^2^ culture plate at 100% confluent. The cell pellet was frozen and thawed several times to help lyse the cell membrane. Then, 500 μL of freshly prepared aqua regia (HNO_3_:HCl = 1:3) was added and left to react overnight. Mice organs and fluids (brain, cerebellum, atrium, ventricles, lungs, liver (two main lobules), aorta, stomach, spleen, intestine, kidneys, urine, blood cells and blood plasma) were frozen and lyophilized. The dried tissue samples were weighed and immersed in 1.2 or 8 mL of fresh aqua regia, depending on the tissue weight. The tissues were then submitted to a microwave digestion process using a Speedwave XPERT Microwave Digestion System (Berghof). Initially the samples were heated to 160 °C at 30 bar with a 10 min ramp, and held for 10 min. Then, the temperature was increased to 190 °C and 30 bar, with a ramp of 5 min and held in these conditions for 20 min. The solution was then cooled to room temperature. Subsequently, the samples were diluted with 2% HNO_3_ and analyzed by inductively coupled plasma mass spectrometry (ICP-MS). All experiments were performed in triplicate. The Au concentration in each sample was determined by iCAP-Q ICP-MS (Thermo Scientific, Bremen, Germany) equipped with an autosampler ASX-520 (Cetac Technologies Inc., NE, USA) (n = 3) and QtegraTM v 2.6 (Themo Scientific).

## Supplementary Material

Supplementary figures.
