# Supplementary material for: Fibroblast-derived extracellular vesicles as trackable efficient transporters of an experimental nanodrug with fibrotic heart and lung targeting
Source: Theranostics. 2024 Jan 1;14(1):176–202. doi: 10.7150/thno.85409 (PMC10750212; doi:10.7150/thno.85409)
Supplement: Supplementary file 1 — Supplementary figures. [file thnov14p0176s1.pdf]

Supplementary Figures

Supplementary Figure 1

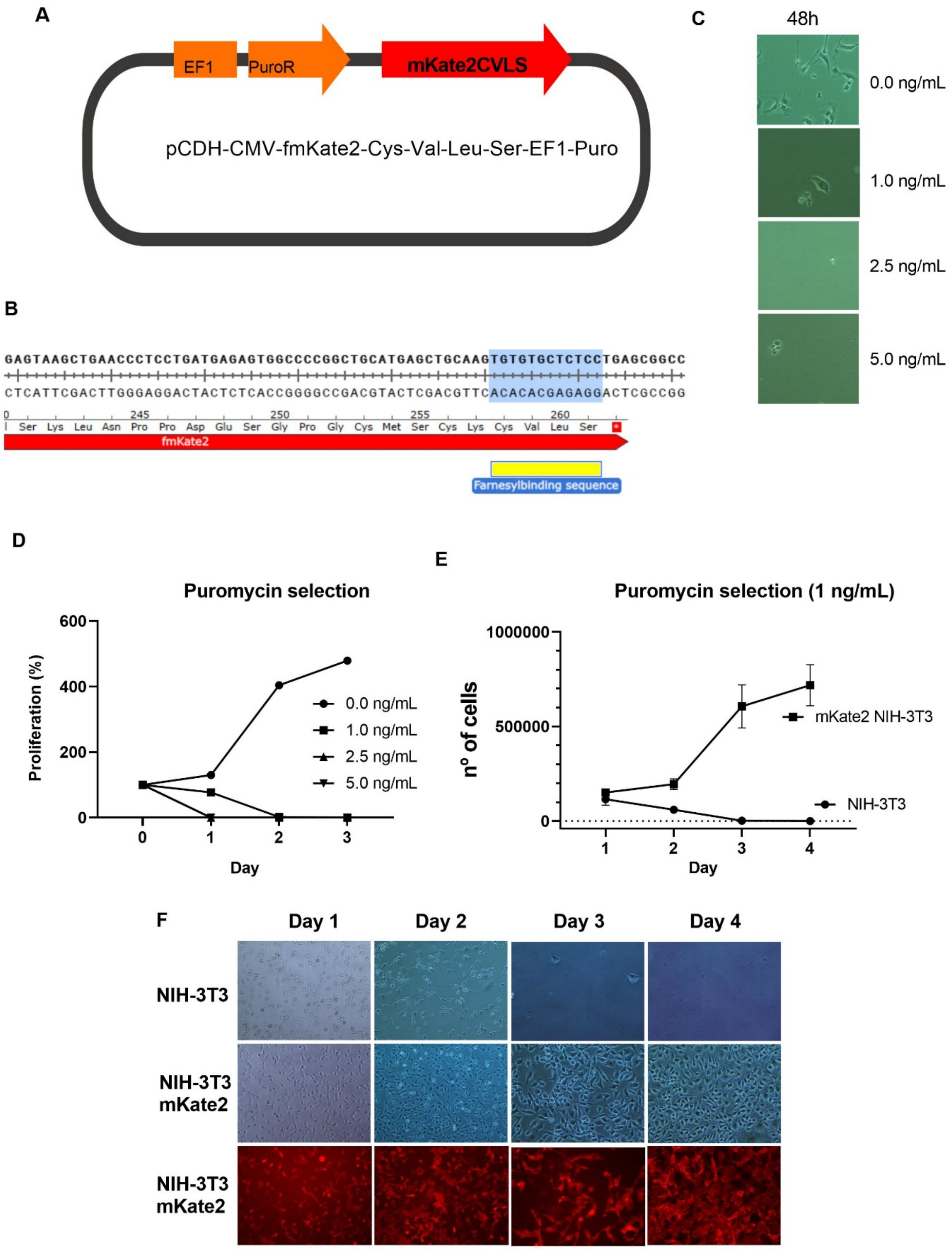

***Supplementary Figure 1: Generation of genetically engineered mKate2 NIH-3T3 fibroblasts.** A) Drawing depicting the mKate2 plasmid transfected to obtain fluorescent NIH-3T3 mKate2 fibroblasts and their derived fluorescent mKate2 EVs. **B**) Detail of the Cys-Val-Leu-Ser sequence fused to the red fluorescent mKate2 peptide to increase its hydrophobicity and thus its membrane localization. **C**) Images showing viability of NIH-3T3 fibroblasts in the presence of puromycin (0.0, 1.0, 2.5 and 5.0 ng/mL. **D**) Graph showing the percentage of NIH-3T3 proliferation in the presence of puromycin (0.0, 1.0, 2.5, and 5.0 ng/mL) over 3 days. **E**) Graph of puromycin (1 ng/mL) selection of NIH-3T3 mKate2 and absence of NIH-3T3 survival over 4 days. **F**) Images of puromycin (1 ng/mL) selection of NIH-3T3 mKate2 and absence of NIH-3T3 survival. The third row shows the viability (red fluorescence) of the newly generated mKate2 NIH-3T3 cell line.*

Supplementary Figure 2

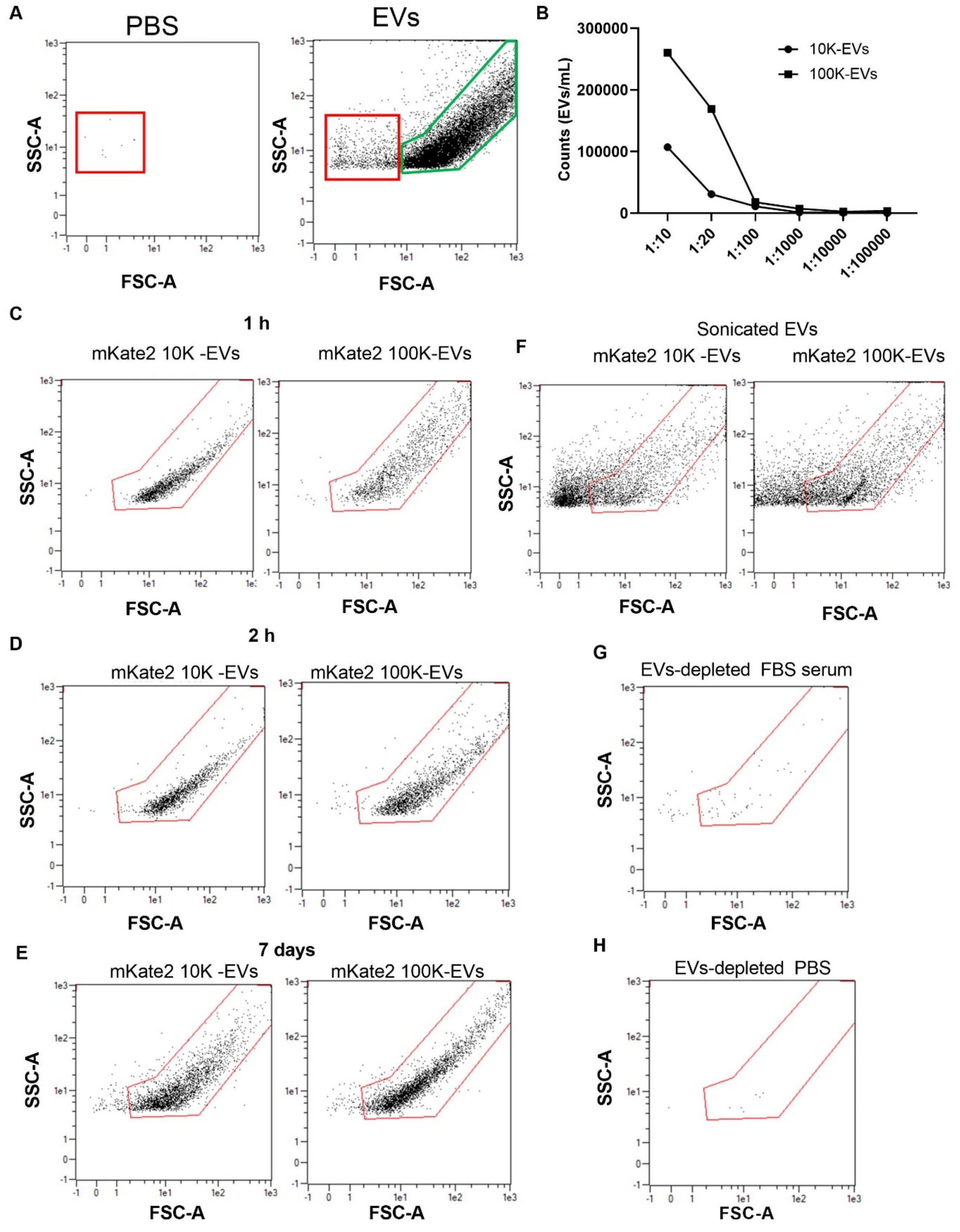

Supplementary Figure 2: Measurements of EVs stability. *A) Representative images of forward scatter (FSC-A) versus side scatter (SSC-A) in the 645 nm fluorescent channel (mKate2) showing PBS background detection (left) to select detection area of mKate2 EVs (right) by flow cytometry.* ***B****) Graph showing the effective signal/sample ratio (1:10 and 1:100) of serial dilutions of mKate2 10K-EVs and mKate2 100K-EVs to observe the possible dilution range for EV measurements starting from a concentration of* $2x10^9$ *EVs.* ***C,D,E,F****) Flow cytometry measurements of mKate2 10K EVs (left*

*panels) and mKate2 100K EVs (right panels) after incubation in EVs-depleted FBS serum at 37 ºC and then resuspended in EVs-depleted PBS; (**C**) 1 h of incubation, (**D**) 2 h of incubation, (**E**) 7 days of incubation (**F**) negative control of broken EVs by sonication. **G**) Flow cytometry measurement of the negative control EVs-depleted FBS serum. **H**) Flow cytometry measurement of the negative control EVs-depleted PBS.*

Supplementary Figure 3

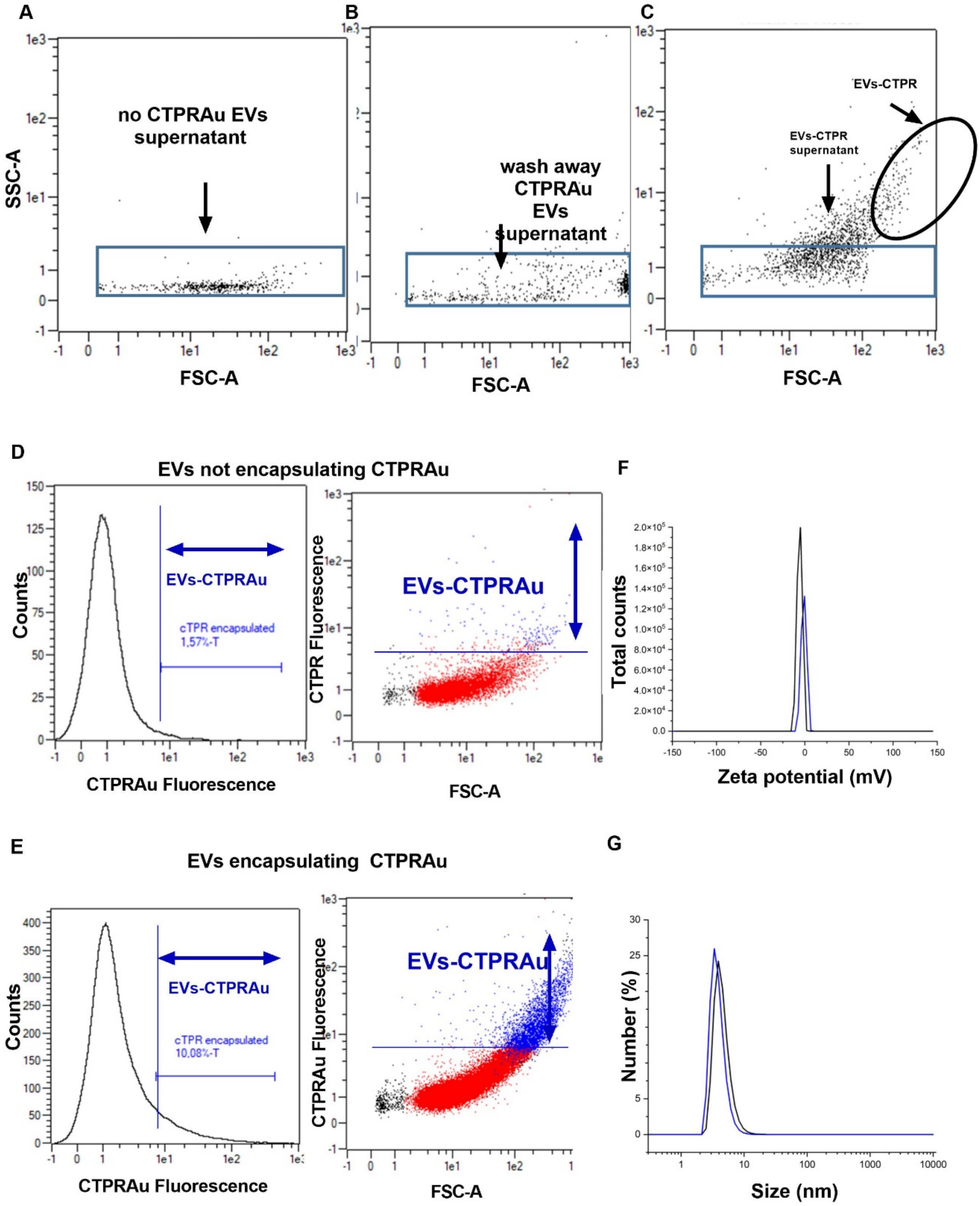

Supplementary Figure 3: Set up for detection of mKate2 EVs-CTPRAu. A) *Delimitation of the supernatant area of unencapsulated EVs by forward scatter (FSC-A) versus side scatter (SSC-A) by flow cytometry.* ***B****) Detection of EVs-CTPRAu supernatant in the delimited area.* ***C****) Definition of the area to find EV-CTPRAu, clearly separated from the area marking the EV-CTPRAu supernatant by flow cytometry.* ***D****) Fluorescence of CTPRAu versus counts and FSC-A in EVS samples that do not encapsulate CTPRAu to differentiate the area of EVS that do not encapsulate CTPRAu from the area of EVs-CTPRAu detection by flow cytometry.* ***E****) Fluorescence of CTPRAu versus counts and FSC-A in EVs-CTPRAu samples to mark the area of their detection by flow cytometry.* ***F****) Zeta potential measurements of CTPRAu.* ***G****) Hydrodynamic diameter of CTPRAu measured by DLS.*

Supplementary Figure 4

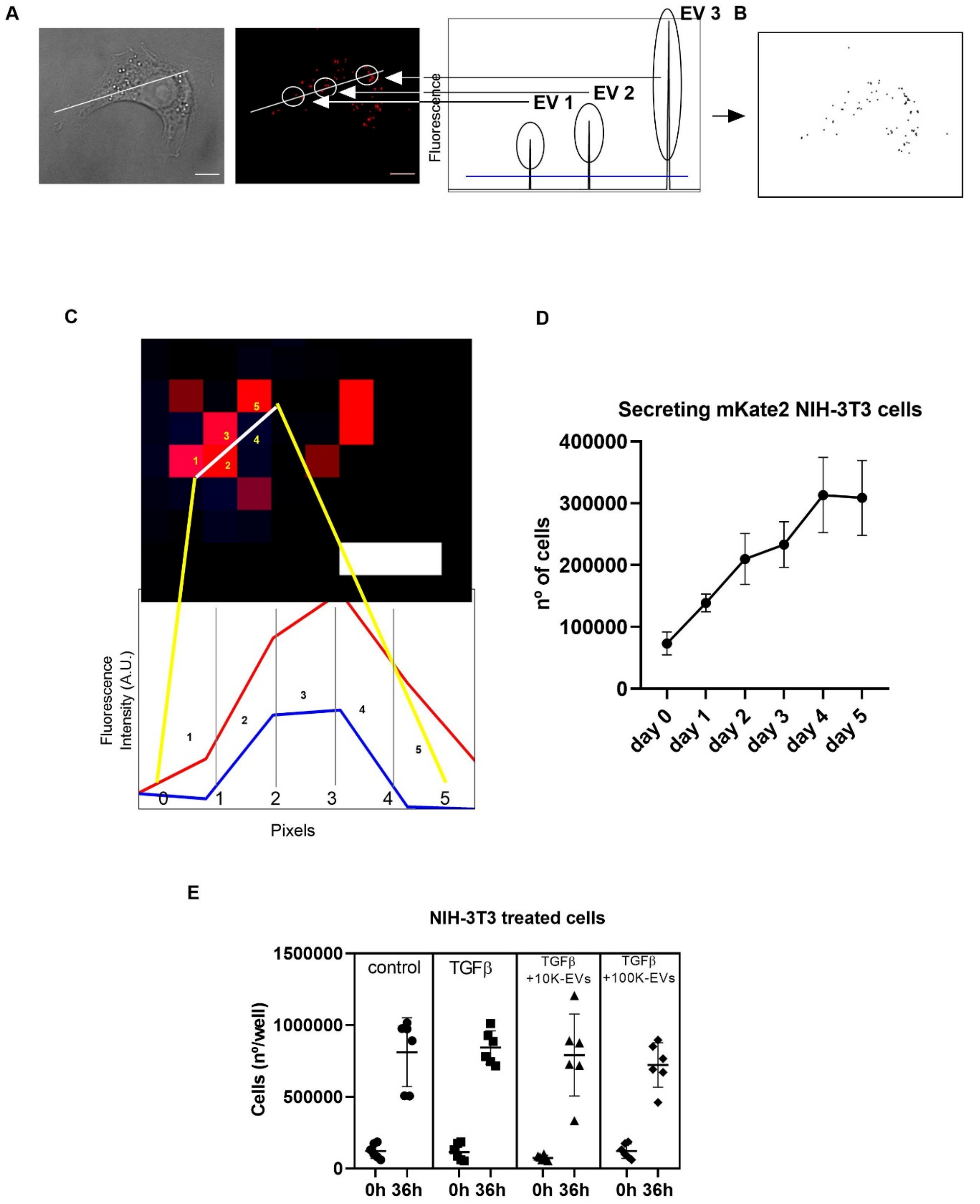

Supplementary Figure 4: Set up for detection of mKate2 EVs-CTPRAu in cells and their viability when are treated or are secreting EVs*. **A)** Sequence of three panels of NIH-3T3 cell images (brightfield, confocal microscopy and selection of EVs (EV1, EV2 and EV3) for measurement of their fluorescence) fixing the backgrownd (blue line of the third panel) to analyze the fluorescence intensity along the white line marked in the first two panels. **B)** Application of ImageJ software filters that allow quantification of EVs and CTPRAu by their fluorescent signal intensity. **C)** Amplification of an EV-CTPRAu to pixelate the image and generate the signal intensity spectra of mKate2 and CTPRAu*

indicating where both fluorescences colocalize within the EV. ***D****) Measurement of the number of live cells during the 5 days of EVs secretion in depleted media n = 3 independent assays.* ***E****) Count of the number of cells per well after EV administration to TGFβ-activated NIH-3T3 cells at 0 and 36 h after treatment, n = 5 independent assays.*

Supplementary Figure 5

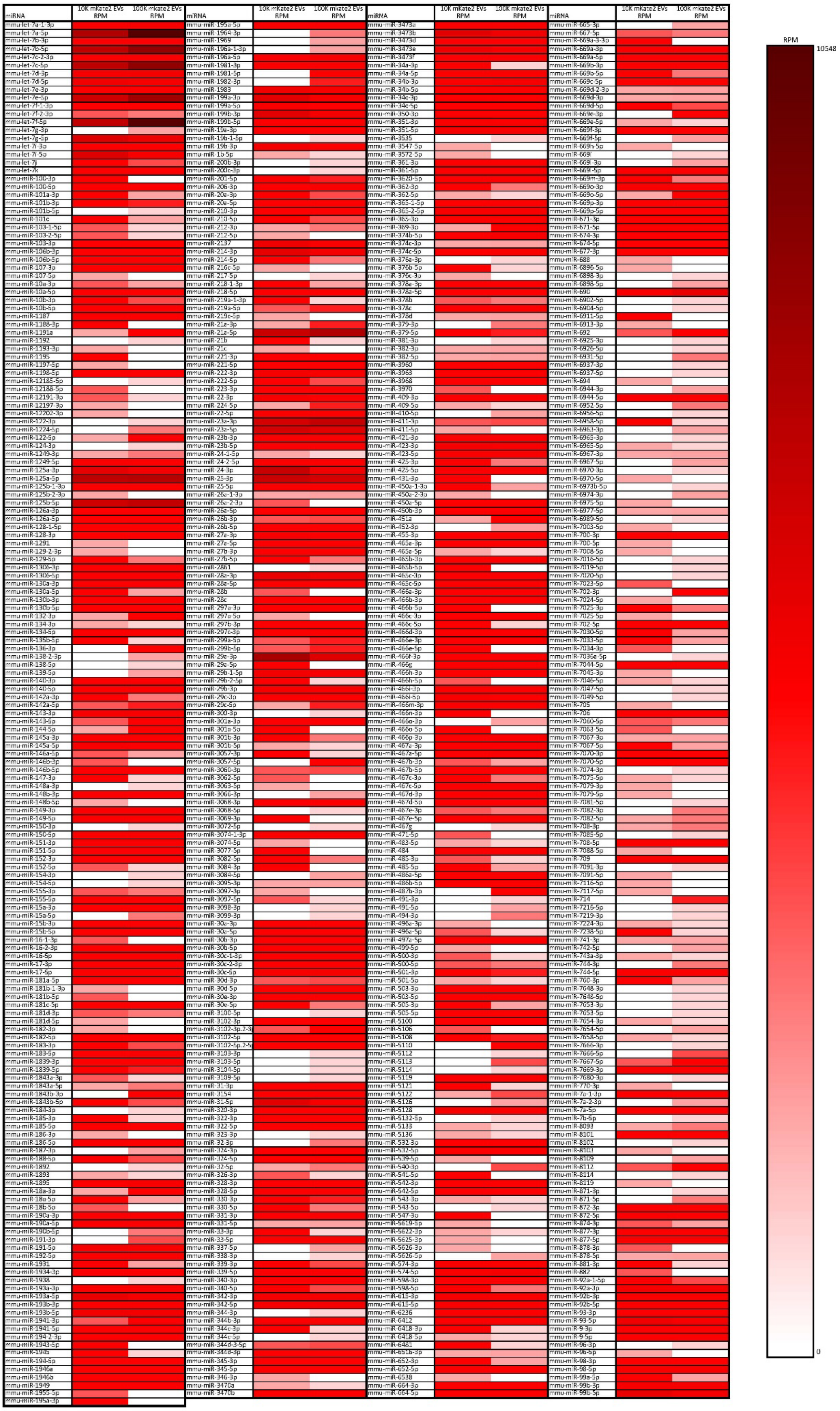
miRNA
10K mKate2 EVs RPM
100K mKate2 EVs RPM
RPM
10548
0

Supplementary Figure 5: The heatmap of identified miRNAs of mKate2 10K-EVs and mKate2 100K-EVs. *Data in Reads Per Million (RPM) was drawn on log 2 normalized read counts in relation to the expression and they were obtained using excel16 program. There are 681 rows and 8 columns corresponding to each miRNA and type of EV (mKate2 10K-EVs left and mKate2 100K-EVs right, respectively. The heat bar indicates with white color the absence of miRNA and with darkest red the maximum RPM obtained (105486).*

Supplementary Figure 6

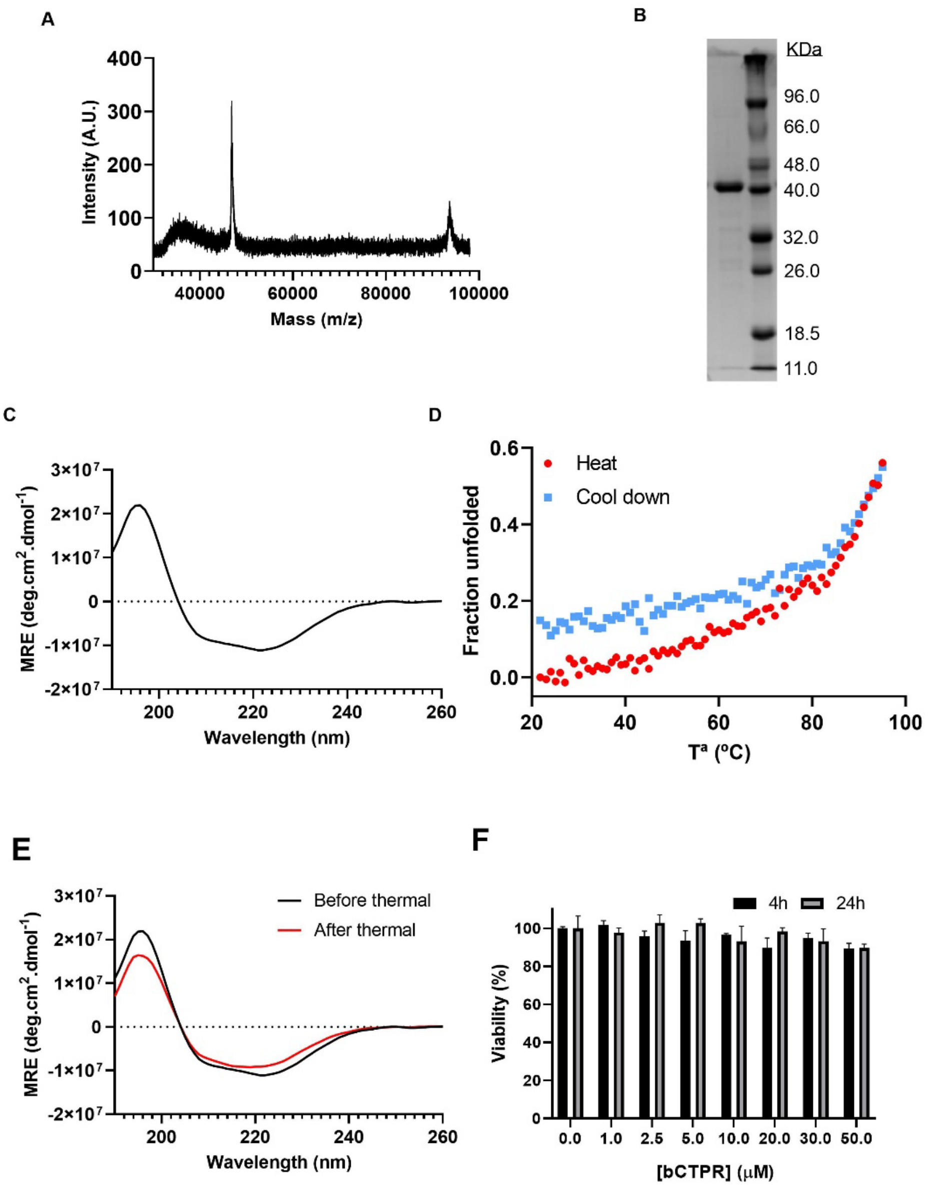

Supplementary Figure 6: bCTPR characterization. *A) MALDI-TOF of bCTPR and **B)** SDS-PAGE acrylamide gel and showing the expression purity and confirming the molecular weight of the newly designed protein, respectively. **C)** CD spectrum of bCTPR showing that the protein is properly folded and has the typical α-helical secondary structure of CTPR proteins. **D)** Thermal denaturation curves of bCTPR. The novel chimeric protein showed to be stable up to 40 ºC, temperature at which it starts losing some structure. **E)** CD spectra recorded before and after thermal denaturation, showing that*

even after exposure to extreme temperatures (95 ºC), the protein is able to refold. ***F)*** *Cell viability of NIH-3T3 fibroblasts incubated with different concentrations of bCTPR for 4 and 24h.*

Supplementary Figure 7

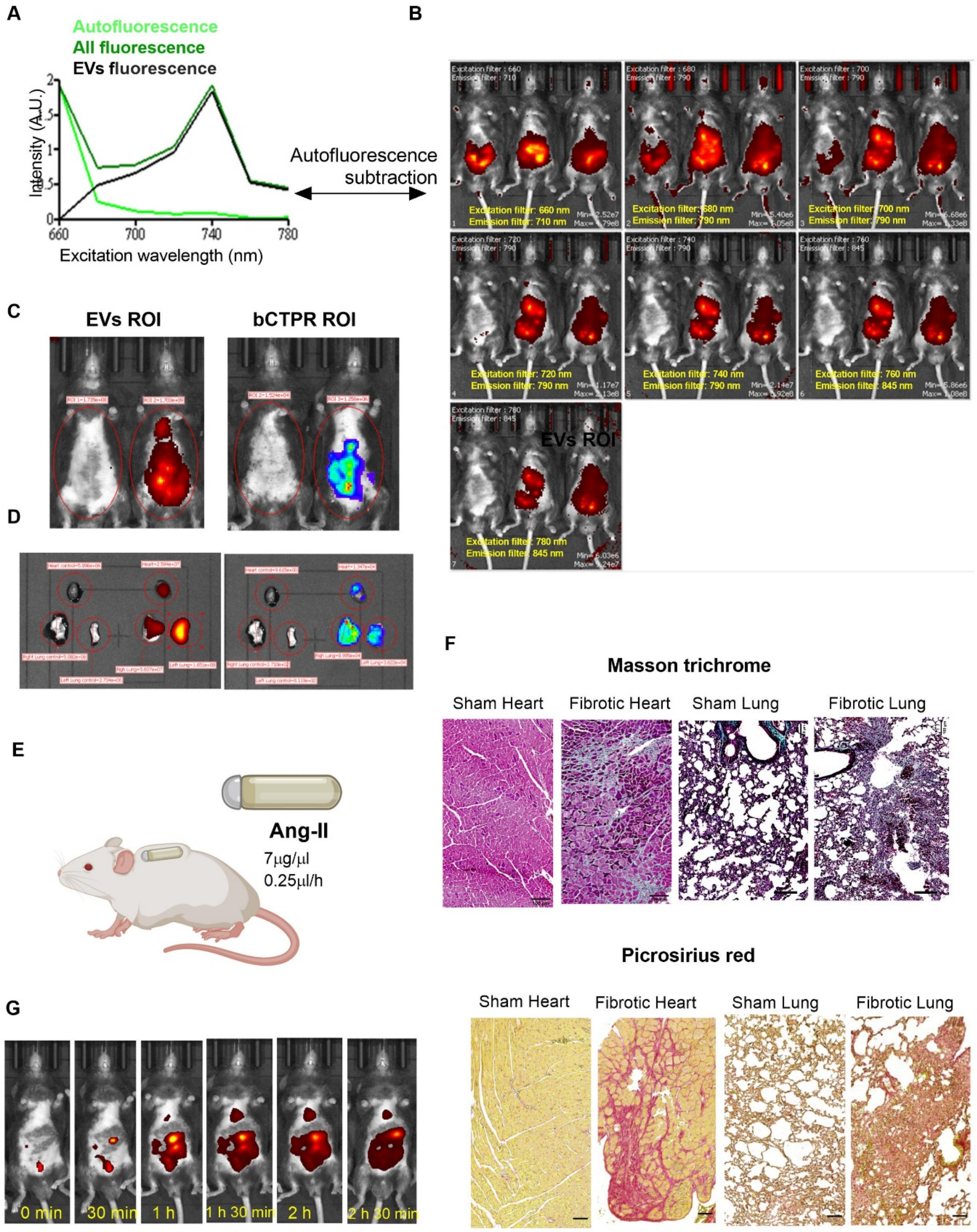

***Supplementary Figure 7: In vivo imaging setup for detection of mKate2 EVs and bCTPR encapsulated in mKate2 EVs in mice**. A) Excitation wavelength spectra covering the 660 nm to 780 nm range of tissue autofluorescence (light green), EVs fluorescence (black) and both signals (dark green). **B**) excitation/emission bundles including the 660 nm to 780 nm range of excitation wavelengths and 710 nm to 845 nm emission wavelengths, plus subsequent subtraction of the autofluorescence background to select the best excitation/emission bundle to detect EVs. **C**) in vivo delineation (abdominal and thoracic areas) of the regions of interest (ROI) where signal intensity (fluorescence for EVs and bioluminescence for bCTPR) will be measured. **D**) Delineation of Regions of Interest (ROI) of ex vivo heart and lungs (selected organs) where signal intensity will be measured (fluorescence for EVs and bioluminescence for bCTPR). **E**) Cartoon depicting the insertion site of the Ang II-loaded osmotic minipump to generate fibrosis in mice. **F**) Masson's trichrome and Picrosirus red images of the heart and lungs of Sham control and Ang II-treated mice. In green (Masson's trichrome) and in red (Picrosirus red) fibrotic areas are detected in Ang II-treated mice. Black bar indicates 100µm. **G**) In vivo visualization of mKate2 EV at 0h, 30 min, 1h, 1h30min, 2h, 2h30min after intraperitoneal injection of mKate2 EV.*
